# Divergence of Ethnobotanical Knowledge of Slovenians on the Edge of the Mediterranean as a Result of Historical, Geographical and Cultural Drivers

**DOI:** 10.3390/plants10102087

**Published:** 2021-10-01

**Authors:** Ivana Vitasović-Kosić, Mitja Kaligarič, Josip Juračak

**Affiliations:** 1 Faculty of Agriculture, University of Zagreb, Svetošimunska cesta 25, 10000 Zagreb, Croatia; jjuracak@agr.hr; 2 Faculty of Natural Sciences and Mathematics, University of Maribor, Koroška cesta 160, 2000 Maribor, Slovenia; mitja.kaligaric@um.si

**Keywords:** North Adriatic Karst, traditional uses, interviews, local knowledge, cultural value, historical heritage

## Abstract

State boundaries limit human contacts in a homogenous context of a landscape and its natural features, including plants. After nine centuries of separation, finally the two territories in Slovenia share the same political history. In this paper we tried to answer the question to which extent the past political borders, geographical and cultural drivers affect today’s traditional knowledge on wild plants use of Slovenians, living unified in the same political entity. Data were collected using 60 in-depth semi-structured interviews, from March to August 2019, in two municipalities: Komen at Karst and Izola in Istria concerning food, medicinal, economic use, and local customs. The results indicate a quite large divergence in ethnobotanical and ecological knowledge between the two studied areas. In the Komen area, many people still use wild plants daily for various purposes (*Taraxacum officinale*, *Melissa officinalis, Urtica dioica, Cornus mas,* and *Sambucus nigra*). In contrast, this is limited to fewer people in the Izola area and mainly to seasonal use of specific plants (*Asparagus acutifolius*, *Rosa canina*, *Salvia officinalis, Foeniculum vulgare* and *Rubus caesius*). Unusual for the Mediterranean is the use of young shoots of *Clematis vitalba*, in the Izola area prepared as omelettes. We can assume that these differences are partly due to minor differences in climatic conditions and partly due to the influence of different cultures and cuisines. In the first place, the impact of Austro-Hungarian eating habits and cuisine can be seen on the area around Komen. Moreover, temporal “layers of knowledge” across the time scale are additionally mixed by the immigration of people from other parts of Slovenia or abroad, or with the influence of local herbal specialists. At last, we conclude Komen area knowledge is alive and homogeneous, and more connected to their local identity.

## 1. Introduction

Per definition, traditional ethnobotanical knowledge has been transmitted from generation to generation by word of mouth for centuries and millennia. In recent times, it is also transferred with written sources or other media sources, which mix the layers of the knowledge in space and time. We may consider ethnobotanical knowledge part of local ecological knowledge, and it can be, but not necessarily, regarded as traditional [1]. Van den Boog et al. [2,3,4] categorised the dynamics of local ecological knowledge transmission into vertical (between generations within the family), horizontal (between people of the same generation), and oblique (between generations not belonging to the same family) in a given territory. It has been found that different drivers affect the evolving dynamics of ethnobotanical knowledge transmission, including political circumstances [1]. 

Over the last few years, cross-border ethnobotany has received increasing attention from [1,2,3], as it is an excellent tool for exploring the effects of different social and political contexts on ecological knowledge, including botanical knowledge. The importance of a political border has been stressed on the medical, veterinary, and wild food ethnobotany of the Hutsuls living on the Romanian and Ukrainian sides of Bukovina [2]. A political borderline limits people’s contacts in a homogenous context of a landscape and its natural features, including plants. In that regard, the horizontal transmission of the knowledge might be substantially limited, and in time the once homogenous knowledge might differ.

The study of Hutsuls in Romania and Ukraine—where the Hutsuls have been split into two political entities for the last 80 years—revealed that ethnobotanical knowledge transmission occurs in different ways despite a common cultural and linguistic background on each side of the border [1]. However, there is no study on to which extent the past political borders affect today’s traditional knowledge of one nation, living unified in the same political entity. Such comparison would enable us to evaluate the magnitude of vertical and horizontal knowledge transmissions. Slovenians were (and still are) split into different political entities over history, so they are very suitable to answer the above-stated question on their ethnobotanical knowledge.

Studies on the use of wild plants are scarce in Slovenia, and only a few ethnobotanical studies have been conducted to date [5]. The regions of Kras (the Classical Kras) and the inner Slovenian Istria are very suitable for such comparison. One ethnobotanical study was performed in the Italian part of the Kras region and north Istria [6], revealing 59 plants used in that territory. Another study, conducted in Italian and Slovenian parts of Kras [7], provided a list of 124 plants used for healing, food, toys, superstitions, and folk traditions. Marinac et al. [8] studied the region of the municipality of Izola, where also ethnobotanical data are partially available. 

An interesting study on the ecological knowledge of Slovenians and Friulans was performed in Italy (Friuli) [3] and revealed a divergence between the two linguistic communities in one environmental space. Namely, food and medicinal uses present remarkable differences between the two communities: they often used the same plants in different recipes, which represent an expression of distinct identities, due in part to “the inner border” and its embedded geographic and cultural isolation [6]. 

From the 9th to the 20th century, the territory of the Slovenian Kras was almost continuously under the rule of Germans and Austrians [9]. The Slovenians in inner north Istria, including the area of today’s Izola, were under the rule of Venice from the 9th to the 18th century. In the 18th century, this area came under the authority of Austria, and since then, after nine centuries of separation, the two territories share the same political history. Only for a short period at the beginning of the 19th century, Istria and the Kras region were under Napoleonic administration. Since the foundation of Austro-Hungarian Empire, the area was under the administration of Austrian Crown as part of Austrian Littoral. At the same time, Trieste and its surroundings had the status of a Free Imperial City [9].

The rule of the Austro-Hungarian monarchy ended after World War I (WW1), when the territory of the studied area became part of the Kingdom of Italy. After World War II (WW2), the area of Komen became a part of Yugoslavia in 1945, while the area around Izola belonged to the Free Territory of Trieste under the Yugoslav administration until 1954. From 1955, both areas were under Socialist Federative Republic Yugoslavia until 1990, when they became an integral part of the present Republic of Slovenia.

Therefore, the situation of Slovenians is just the opposite as to the status of Hutsuls in Romania and Ukraine [1]. In that light, this study’s general aim was to explore ethnobotanical knowledge’s common and differential traits in two geographically close regions of the same nationality, unified in the same political entities for the last 200 years but split apart for 1000 years before that.

In particular, we also aimed to reveal the extent of “old” traditional knowledge vs. “new” knowledge, perhaps due to homogenisation in recent times. Since the traditional use of wild plants has never been fully documented, this study focuses on edible and medicinal plants, plants used for cosmetic, religious or traditional ceremonies, tools, and veterinary medicine.

## 2. Materials and Methods

### 2.1. Study Site

**Figure 1:**
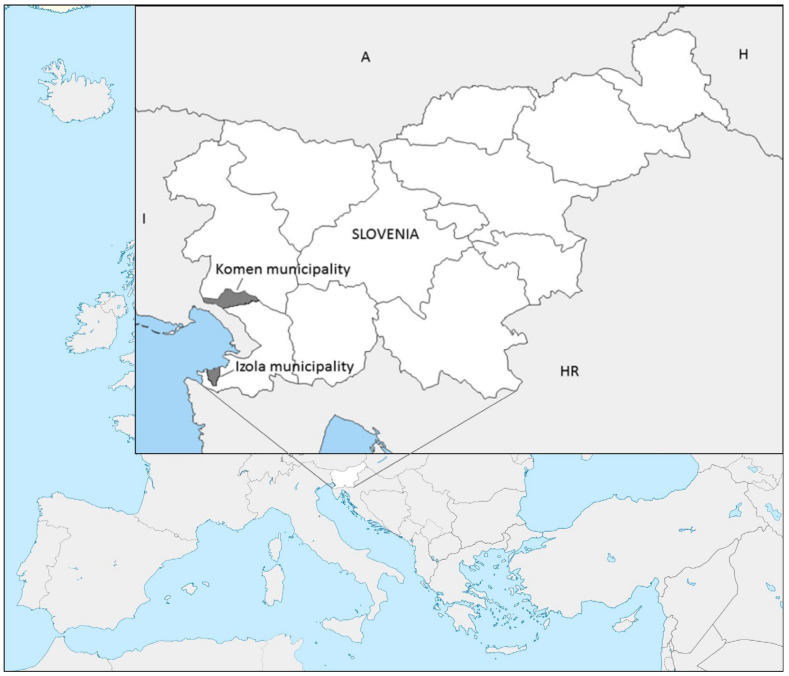
The geographical position of the study site.

The field research was conducted in the Slovenian Coastal-Karst region in two municipalities: Komen and Izola (Figure 1). Komen is located on the Karst Plateau, while Izola is in the northern part of the Istria peninsula.

The following villages in the Komen municipality were included in the survey: Štanjel, Kobjeglava, Škrbina, Štorje (Sežana), Tomačevica, Tupelče, and Volčji grad. This is a typical Sub-Mediterranean area, ranging between 400 and 500 metres of altitude, covered initially with thermophilous forests with dominant *Quercus pubescens*, *Ostrya carpiniflia*, *Fraxinus ornus*, and *Acer campestre*, but deforested already in Roman times, with a peak in the Middle Ages. The prevalent land use was extensive pastures, while in the dolinas (i.e., valleys), people established meadows, vineyards, and fields. In the last 100 years, large-scale spontaneous reforestation occurred: 80% of pastures turned into scrub and pioneer woodland. Only 20% of ex-grasslands remained in a good state [10]. The local flora is a mixture of Sub-Mediterranean-Illyrian, Central European, Mediterranean, and temperate geo elements.

The municipality of Izola consists of the city of Izola and hilly hinterland with villages Cetore, Malija, Nožed, Korte, Šared. Since a far more autochthonous population is in the hinterland, we covered the settlements mentioned above with interviews only. The area is flysch sandstone, covered initially with thermophilous oak-dominated forests. Forests have almost the same composition as in the Komen area, but with more Mediterranean elements in the understory. Due to fertile soil, people converted forests to fields, vineyards, olive groves, and grasslands, which used to be terraced, now consolidated in larger plots for easier cultivation. The abandonment of cultivated land here occurred in the late 1950s and 1960s, but it has been re-cultivated substantially in the last two decades [10]. The flora is a mixture of sub-Mediterranean Illyrian, EU-Mediterranean, Central European, and temperate species. 

We can conclude that studied areas are pretty similar in climate and vegetation, with some differences regarding geological and hydrological conditions.

### 2.2. Sampling and Interviews

We conducted the research in line with the American Anthropological Association Code of Ethics [11] and the International Society of Ethnobiology Code of Ethics [12]. We interviewed local people from two municipalities in the settlements mentioned above using the snowball sampling technique. The informants were chosen from people who were born in the investigated area and had their ancestry there.

Data were collected using in-depth semi-structured interviews and the free listing method, accompanied by informal walks (and talks) with selected informants, from March to August 2019. We also asked for a recommendation for crucial informants, i.e., the most knowledgeable people in the village.

Participants were approached outside by walks around the places or during their farm work, where the respondents gathered plants and could identify the supplied names. We asked for information on folk names, ways of use, parts used, and preparation methods for each taxon.

Altogether, we obtained data from 60 interviews involving 65 local informants (55 single informant interviews and five interviews involving two people). The mean age of respondents was 67.9 (median 68, minimum 42, maximum 86, 64.62% were female, 35.38% male). The average age of respondents in the Izola area is four years higher than in the Komen area, but this difference is not statistically significant (t-stat = 1.6202, *p* ≤ 0.1102).

Discussions concerned various aspects of the use of plants and their parts that respondents practice or have heard of from food use, through medicinal use, to use in the household, on the farm, or in local customs. Notes were kept on specific uses and recipes for making herbal preparations and dishes.

Plants were identified using standard floras available in this area of Europe, including Mala flora Slovenije [13], Nikolić’s guide for the identification of Flora of Croatia [14], Pignatti’s Flora of Italy [15] and the Flora Croatica Database [16]. Plant names were updated to be consistent with the Plant List [17]. Voucher specimens were collected in the field, usually with the assistance of the respondent, deposited and digitised in the ZAGR Herbarium of the Faculty of Agriculture University of Zagreb.

### 2.3. Data Analysis

The collected data in the form of notes on paper were entered into MS Excel for computational processing. The data set includes the following information: location of the survey, respondent, known or used plants, and how to prepare the plant for use. Each record describes one taxon mentioned by a specific interviewee through several variables: common name, scientific name, use category, parts used, and the way of preparation. The variable use category has seven available non-exclusive modalities: food or drink, alcoholic beverage, medicine, as a tool, animal feed, ceremonial use, and others. 

The computational analysis included only taxa mentioned two or more times, while the qualitative analysis counted in plants noted once if the information about them is relevant to the research.

In the first part of the analysis, the absolute and relative frequencies of the recorded plant taxa were calculated. The Relative Frequency of Citation (RFC) was calculated using the following formula [18]:

RFC = FC/N,(1)

where FC is the absolute frequency or number of mentions of a single taxon and N is the number of informants in total or in a single area.

The importance of a single taxon for the overall study and separate survey areas was assessed using Smith’s S saliency measure [19]:

Sj = ((L − Rj + 1)/L)/N, (2)

where *L* is the length of the list per informant, R_j_ is the rank of taxon j in the list, and N is the number of lists in the survey. This indicator allows identifying items that are typical in the research area, taking into account the frequency and order of citation.

The degree of correspondence or similarity between the Izola and Komen areas regarding the plant species recorded and their categories of use was measured using the Jaccard Similarity Index (JI). The similarity index for areas A and B within a single survey is calculated using the following expression:

JI = C/(A + B−C) × 100(3)

In this case, C is the number of taxa common to both areas, A is the number of taxa recorded in area A, and B is the total number of taxa in area *B* [1]. We adapted the formula to calculate the similarity of the plant uses by replacing the number of taxa (A and B) with the frequencies of a given use category.

The importance of each taxon to the local community was assessed using the Cultural Value Coefficient (*CV*). The *CV* quantifies benefits to the local population based on the seven plant use categories: eating or drinking (E), production of alcoholic beverages (A), medicinal use (M), use as a tool (T), ceremonial use (C), animal feed (AF), and other types of use (O). The *CV* was calculated for all taxa mentioned two or more times at the level of the whole sample (both areas) and by areas studied. We calculated the value of the coefficient according to the following formula:

(4) $${CV}_{e}{=Uc}_{e}\cdot {Ic}_{e}\cdot \sum {IUc}_{e}$$

where *Uc_e_* is the number of reported uses for taxon e divided by the number of use categories (7). *Ic_e_* expresses the number of informants who listed the taxon *e* as useful divided by the total number of informants. *IUc_e_* is the number of informants who mentioned each use of the taxon *e* divided by the total number of informants [20].

The Informant Consensus Factor (Fic) developed by Trotter and Logan [21,22] was calculated to rank respondents’ consensus by individual types of use. This indicator is based on the ranking of taxons according to the number of use reports:

Fic = (n_ur_−n_taxa_)/(n_ur_−1), (5)

where n_ur_ represents the number of use reports for a given use category and n_taxa_ represents the number of taxa. The use report indicator (UR) per species is the number of times a particular plant species is mentioned in all use categories [23].

Indicators were calculated using MS Excel and R packages “AnthroTools” [24] and “ethnobotanyR” [25]. As purposive sampling was used to select informants, the statistical analysis results are limited to the population studied.

## 3. Results

The respondents mentioned a total of 199 taxa, of which 157 were mentioned at least twice. There are 50 taxa that were mentioned at least 10 times, 21 were mentioned 20 times or more, and 14 have a mention frequency of 25 or more. In the Komen area, 147 plants with a frequency of two or more were recorded, and in the Izola area, 119 (Table A1). The difference of 28 taxa indicates that in the Komen area the level of knowledge about wild plants is higher than in the Izola area.

The number of taxa cited per questionnaire is lower in the Izola area than in the Komen area. The overall average for 60 questionnaires is 25.15 taxa per respondent, with the mean for Komen (28.63) being significantly higher than the average for Izola (21.67) (t-stat = 2.432, *p* ≤ 0.018). The highest number of taxa per interview is 76 (Komen), and the lowest is 10 (Izola). The Komen area occupies the northern part of Slovenian Karst, where Lumpert and Kreft [5] recorded 77 species in a sample of 25 respondents investigating the use of plants for medicinal purposes. On average, 20 different plant species were recorded per respondent. The lower number of species recorded in Lumpert and Kreft study [5] is probably due to their focus on plants for medicinal use.

The taxa with the highest relative frequency of citation (RFC) are *Taraxacum officinale* (RFC = 0.88), *Asparagus acutifolius* (RFC = 0.85), *Rosa canina* and *Foeniculum vulgare* (RFC = 0.63), and *Urtica dioica* (RFC = 0.62). Seven out of 10 species with the highest overall RFC were found in both studied areas. RFC values are higher in Komen area for 6 of the 10 taxa, while RFCs are higher in Izola hinterland for *Asparagus acutifolius*, *Rosa canina*, *Salvia officinalis*, and *Rubus caesius*. However, there are some differences between the areas in the RFC rank of taxa (Table 1). 

**Figure 2:**
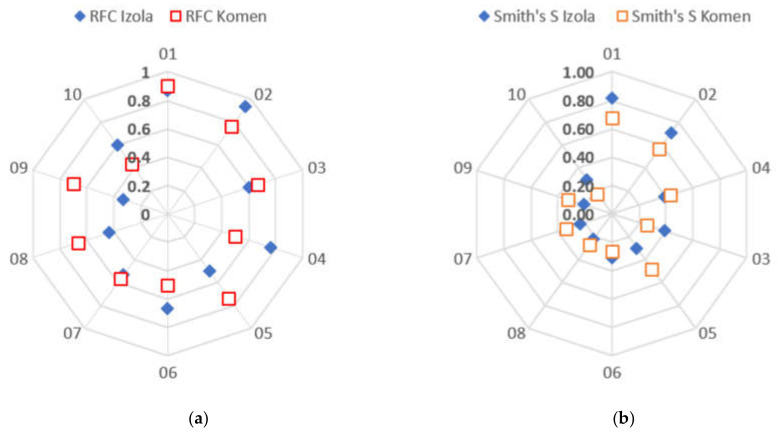
(**a**) RFC for plant taxa* with RFC >= 0.5 by survey area, (**b**) Smith’s S for plant taxa* with RFC ≥ 0.5 by survey area. *01—*Taraxacum officinale*; 02—*Asparagus acutifolius*; 03—*Foeniculum vulgare*; 04—*Rosa canina*; 05—*Urtica dioica*, 06—*Salvia officinalis*; 07— *Laurus nobilis*; 08—*Sambucus nigra*; 09—*Cornus mas*; 10—*Rubus caesius*.

Out of 157 taxa, 109 are common in both areas, giving a JI = 69.43%. Thus, slightly more than two-thirds of the recorded taxa are shared between the studied areas. Taking 50% or 79 of the most frequently cited taxa JI increases to 92.41%, and for the 20% (31) of the most frequently cited plants JI = 100%. The differences in frequencies between the areas are pretty significant for individual taxa. Among plants with RF ≥ 0.5 (10 of them), the most considerable differences have *Rosa canina*, *Urtica dioica*, *Sambucus nigra*, and especially *Cornus mas* (Figure 2a). 

According to Smith’s S Salience Index (S), in the first two places in both regions are *Taraxacum officinale* (Izola S = 0.816, Komen S = 0.680) and *Asparagus acutifolius* (Izola S = 0.709, Komen S = 0.567). The third most important plant near Izola is *Rosa canina* (S = 0.391) and in the area of Komen *Melissa officinalis* (S = 0.512). The biggest difference in the value of S for plants that have RFC ≥ 0.5 is for the species *Urtica dioica* (Izola S = 0.300, Komen S = 0.478) (Figure 2b).

Most collected and plant parts are aerial parts. The most commonly used part of the plant is the leaf, the use of which is reported in 49% of the 157 taxa recorded. This is followed by flower (21%) and aerial parts (18%). The use of seed is mentioned in 6% of plants, and other parts are used in less than 5% of cases (shoots, taproots, bulb, stalk, cell sup, and bark). 

### Ways of Use

The most common type or category of use is for food or drink, and it is listed for 131 taxa. The second most common use category is medicinal (72 taxa), followed by use for making of alcoholic drinks (53), as animal feed (19), for tools (8), and ceremonial purposes (5 taxa). The use category “Others” was recorded for 37 taxa. The proportion of taxa with use type E (edible) is higher in the Izola area (87%) than in the Komen area (78%). On the other hand, the proportions of plants with use categories A (alcohol), T (tools), AF (animal feed), and O (other) are higher in the area of Komen than in the area of Izola.

This maximum possible number of use categories per taxon is 7, while the highest recorded number was 5 for two taxa: *Lavandula angustifolia* and *Olea europaea*. The majority of taxa (111) have 1 or 2 indicated use categories cited. The average number of uses per taxon is higher for the Komen area (1.74) than for the Izola area (1.26). This difference is statistically significant (t-stat = −4.150, *p* ≤ 0.000). Plants with the highest number of use categories in the Komen area are *Robinia pseudoacacia* (5 use types) and 10 other species with the four use categories per plant (e.g., *Lavandula angustifolia*, *Vitis vinifera*, *Foeniculum vulgare*, *Sambucus nigra*, and *Urtica dioica*). In the Izola hinterland, the plants with the highest number of use types (4) are *Lavandula angustifolia*, *Olea europaea*, *Rosmarinus officinalis*, and *Vitis vinifera*. 

**Figure 3:**
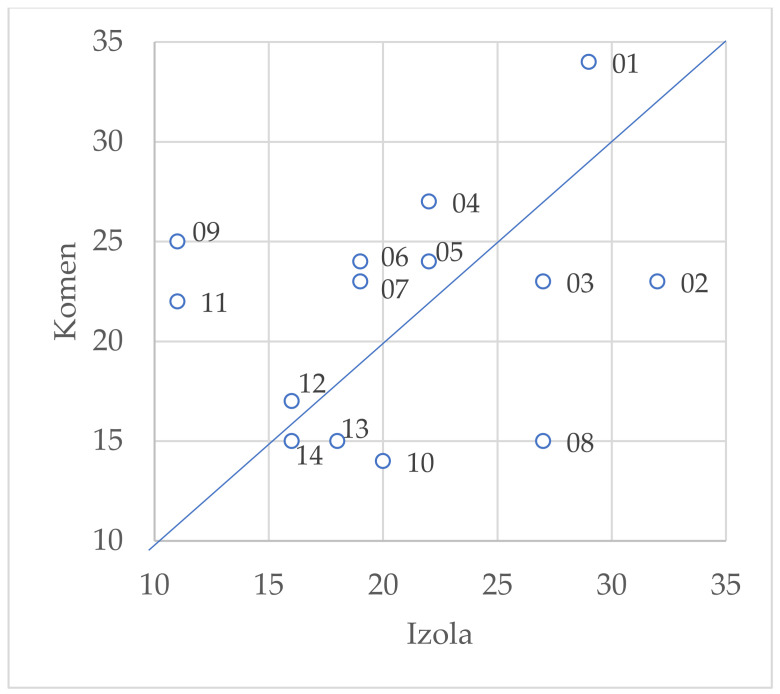
Plant taxa with use reports (UR) > 30 by study areas. Legend: 01—*Taraxacum officinale*; 02—*Asparagus acutifolius*; 03—*Salvia officinalis*; 04—*Urtica dioica*; 05—*Foeniculum vulgare*; 06—*Laurus nobilis*; 07—*Sambucus nigra*; 08—*Rosa canina*; 09—*Cornus mas*; 10—*Rubus caesius*; 11—*Juniperus communis*; 12—*Plantago lanceolata*; 13—*Ruscus aculeatus*; 14—*Vitis vinifera*.

The same plants that have the highest frequencies of citation have the highest frequencies of use expressed in the UR: *Taraxacum officinale* (UR = 53), *Asparagus acutifolius* (UR = 51), *Rosa canina* (UR = 38), and *Foeniculum vulgare* (UR = 38). However, we found differences in UR between the Izola area and the Komen area for specific high-frequency taxa. Examples of significant differences include *Cornus mas* (Komen UR = 25, Izola UR = 11) and *Rosa canina* (Komen UR = 15, Izola UR = 27). For a comparative overview of the UR at the subsample level for taxa with UR ≥ 30, see Figure 3. Points further from the diagonal represent UR pairs with larger differences.

The similarity rating of the studied areas in terms of plant use categories was measured using JI. The results show that the areas are most similar in use category E (JI = 67.18%). They are less similar in use categories M (JI = 41.67%) and A (JI = 30.19%). The biggest differences between regions are in the use categories 0 (JI < 30%) and C (JI = 0%).

**Figure 4:**
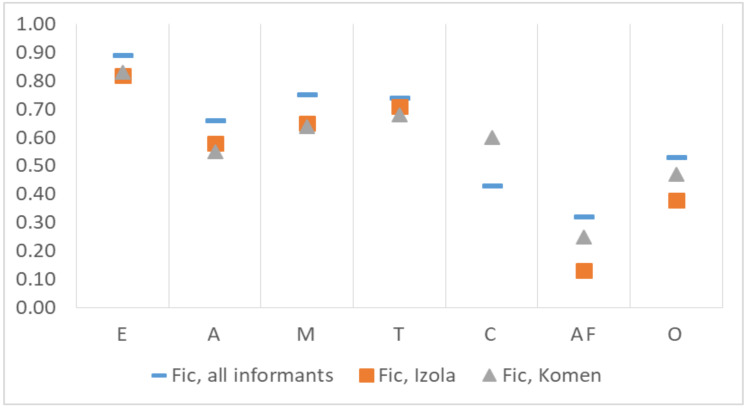
Informant consensus factors for the use categories of plants. E—food or drink; A—alcoholic drinks; M—medicinal use; T—tool; C—ceremonial use; AF—animal feed; O—other not specified ways of use.

The highest consensus among respondents, as measured by the Informant Consensus Factor (Fic), was found for plant use category E (Fic = 0.89) (Figure 4), with negligible difference between the two subsamples. High consensus factors were also found for use category M (Fic = 0.75), with no significant differences between Komen and Izola. 

Fic factors are also higher than 0.5 for use categories T (Fic = 0.74) and A (Fic = 0.66). The indicator is 4% higher for T and 6% higher for A in the Izola area than in the Komen area. The most notable differences between the areas are recorded for the use categories with a low mention frequency (O, AF). 

Table 2 provides a comparative overview of the five most frequently mentioned taxa according to different use categories. Although individual plants repeatedly occur in both study areas for the same use categories, we note specific differences. These differences are more remarkable for use categories E and T than for categories M and A. For example, in category E only two plants are common in both study areas, whereas in category A four are common. In addition to the differences in plant species, we also note differences in the ranks of species by frequency.

At the level of the whole sample, the values of the Cultural Value Coefficient (CV) are highest for *Taraxacum officinale* (CV = 0.379), *Asparagus acutifolius* (CV = 0.334) and *Urtica dioica* (0.282). However, there are differences in the ranks of the highest CV by study area (Table 3).

**Figure 5:**
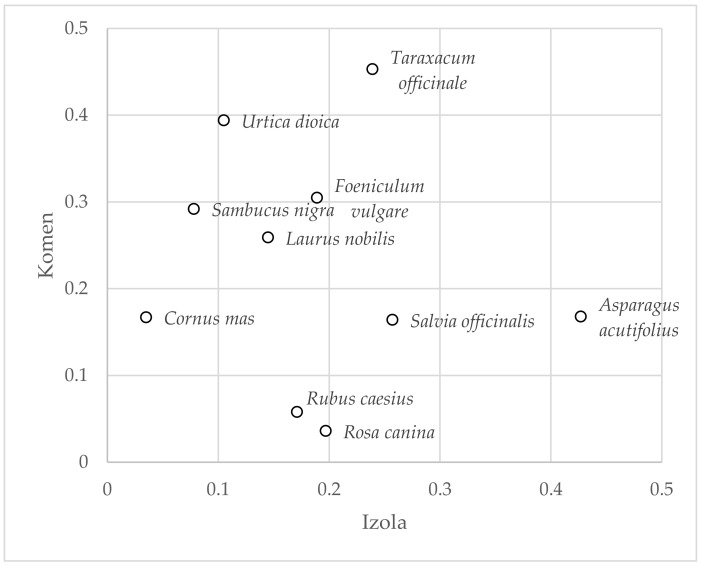
The top 10 plant taxa according to CV and study area.

It can be seen from the Figure 5 that the greatest differences between the research areas have *Asparagus acutifolius*, *Urtica doica*, *Cornus mas*, *Rosa canina*, and *Sambucus nigra* (Figure 5). An overview of the CV values for all plant taxa is available in the Table A2.

## 4. Discussion

Although the traditional use of wild edibles is largely decreasing due to socioeconomic and ecological changes over Europe, wild plants are becoming a part of the new thinking about food. They become very important in healthy food, food security and slow food movements [26]. The importance of studying the relationship between the human community and its surrounding wild plants from a sociological perspective has long been noted and emphasised. As early as 1932, Gilmore [27] emphasised the role of ethnobotany in studying the socioeconomic characteristics of communities and their development, both from an economic and a cultural history perspective. It is important to remember that ethnobotanical knowledge in a community also changes over time under the influence of environmental, social, economic, and political changes [28,29].

### 4.1. Differences between Localities of Komen (Karst) and Hinterland of Izola (Istria) 

The local population in the Komen (Karst) area has always lived more in contact with nature, and ceremonial and traditional customs are still present in everyday life. Over the decades, wild plants have been used for edible and medicinal purposes, to make alcoholic drinks, cosmetics, hand tools, furniture, folk instruments, decoration and animal feed. The population in the hinterland of Izola (Istria) have used the wild plants much less and primarily only for edible and medicinal purposes. The people of the Izola area have focused more on the cultivation of traditionally naturalized plants and live mainly from a sea-based economy (fishing and fishing industry) and tourism. We found that wild plants are primarily used as medicinal herbs and spices, and *Olea europaea* is the only plant with all seven categories of use.

A close resemblance to the Croatian part of Istria is evident in the hinterland of Izola in many local names and uses of plant species [30]. This similarity is expected since it is the same geographical region that used to be within the same state until 1990.

However, the situation is somehow different in the Komen area. Botanical tradition is deep-rooted in that area. A well-known botanist and physician Pietro Andrea Mattioli lived in Gorizia, only 30 kilometres from Komen. His commentaries on the “De Materia Medica of Dioscorides” (first published in Italian in 1554) were translated from Latin into several languages by the end of the 16th century and were a high point of botanical knowledge of the time, including plant medicine. Many plants from the Kras area were described in Mattioli’s work for the first time. Several books printed in German on wild plants and their use in food and medicine date from the 19th century. These books are also important references in today’s publications on botany and the use of wild plants. Interestingly, the memories of the inhabitants of Komen about the reign Austro-Hungarian are positive. That is related to the processes of liberalisation, political change, industrialisation and development at that time, especially around Trieste [31].

The Slovene population of the villages in the hinterland of Izola was for centuries in contact with the Italian-speaking population of the towns of Izola and Koper through the economic exchange (sale of products, employment in factories) and administrative matters. They were almost all at least partially bilingual [8]. Their botanical knowledge was based almost exclusively on traditional knowledge.

The unifying factor for the pharmacopoeias must be that the two researched localities do not have significantly different climates and vegetation. The vegetation is mainly composed of typical sub-Mediterranean maquis, agricultural land, and *Quercus pubescens* forests. Nevertheless, the Komen area is more influenced by a mixture of sub-Mediterranean and continental climates, while around Izola more Mediterranean influence is present. We can confirm the more substantial impact with plants having RF=0.5 or more: the largest differences are for typical “continental” species like *Rosa canina*, *Urtica dioica*, *Sambucus nigra*, and especially *Cornus mas*. Those have a significantly higher presence in Komen area (Figure 2). Species *Urtica dioica* has the biggest difference in the value of S between study areas. It has a significantly higher presence in Komen area, which we expected since it is a widespread plant in the continental part of Europe [32].

Interestingly, six of the top 10 taxa are used as food in omelette preparation, but some plant taxa are used in only one area. Omelettes, locally named “frtalja” in the Komen area and “fritaja” in the Izola area, are made with *Asparagus acutifolius*, *Foeniculum vulgare*, *Urtica dioica*, *Melissa officinalis*, *Ruscus aculeatus*, and *Dioscorea communis*. *Mellisa officinalis* is more widespread in the Komen area, while the inhabitants of the Izola hinterland use *Urtica dioica* in omelettes. Use of *U. dioica* is due to the influence of immigrants from more distant land areas. Unusual use of *Tanacetum parthenium* for food was recorded in the Komen area. The leaves of this plant, which is otherwise used for medicinal purposes, are traditionally in the making of omelettes. Lumpert and Kreft [5] also recorded this in their study. A particular feature of local gastronomy in the Komen area is an omelette with *Tanacetum parthenium* and *Melissa officinalis*. On the first of May, a special tradition is to eat an omelette with *Foeniculum vulgare* and *Melissa officinalis* so that mosquitoes will not bite you all season. Although *M. officinalis* and *F. vulgare* originate from southern Europe and the Mediterranean, only *Foeniculum* was already known to the ancient Romans and is still used in the Izola area for “fritaja” and “maneštra” or “mineštra”. On the other hand, Melissa was not known and is still not used in the same area for omelettes. The origin of this habit is unknown, but it is probably a remnant of a wide variety of egg dishes, which tried to be enriched with plants. The plants served as a source of flaxes and vitamins when there was no meat at the end of winter or in times of famine caused by various factors such as war, crop failure, population imbalance or inflation.

The local word for omelettes derives from the Italian word “frittata”. In the area of Komen, this term was changed to “frtalja”, while in the Izola area to “fritaja”. This linguistic divergence perfectly reflects the impact of the historical borders between the Habsburg Empire and the Republic of Venice. As very well known, the plant names represent one of the oldest names, which have the same roots in many different Slavic languages, but they vary slightly from region to region, from language to language, from dialect to dialect, sometimes referring even to different plants. Therefore, also in our case, folk names vary in two researched areas, but not substantially. The influence of Italian language is more evident in the area of Izola.

“Local specialities”, of course, reflect rationality in consumption of high nutrient-value products such as cultivated vegetables, potato and meat. The wild plants enrich and supplement the food produced on farms but cannot replace it. In times of famine, high-quality meat was sold to provide money for basic foodstuffs like flour, salt, sugar or rice. A traditional local dish in the Izola vicinity is minestrone beans: stew dish with *Foeniculum vulgare* and dried, usually less valuable meat parts. Another traditional dish with beans, dried meat and fennel (leaves only) is a goulash like dish, prepared thickly (with flour). 

In the cake category, *Juglans regia* is traditionally used in both areas (the local cake name is “orehova potica”). Some people in the Komen area use *Allium ursinum* and *Artemisia dracunculus* in traditional “potica”. The use of *A*. *dracunculus* is probably a new practice introduced from central and eastern Slovenia, where it is very popular. It has spread as far as Trieste, where it is considered a traditional local pastry under the name “putizza”. In addition, pancake mixtures with the invasive *Robinia pseudoacacia* have been documented in both areas and *Viola* sp. only in the Izola area.

Another use, rare and unusual in the Mediterranean, is young shoots of *Clematis vitalba*, which are used in omelettes and are found only in the Izola area. It is interesting because all species of the genus *Clematis* are known to contain protoanemonin, which irritates the walls of the digestive tract and the skin [33]. *C. vitalba* is the only *Clematis* species used (after cooking) as food. At present, it is less frequently used as food than in the past on the island of Krk [34] and by the ancient South-Slavic diaspora in southeastern Italy [35].

*Rosa canina* and *Salvia officinalis* have been frequently mentioned in other ethnobotanical studies conducted in Slovenia [5]. *Salvia officinalis* is rare in nature in the Kras area, but it is widely planted and grown in gardens and backyards.

*Salicornia perennans* subsp. *perennans*, a plant growing near the sea, is commonly used to be boiled as a side dish in fish meal recipes in the Apulia area (Italy). In our study, *Salicornia* was mentioned twice with instructions that it should be blanched and served with vinegar and olive oil on salad. According to Biscotti et al. [36], interest in *Salicornia* sp. has risen dramatically recently, especially in the Gargano area. It is possible to buy products based on it on the roadsides and in the local markets. This practice was recorded only in the narrow area, indicating the loss of traditional knowledge in other places along the Adriatic Sea.

Another rarity is the traditional use of *Stellaria media* in fresh salad dishes and as a chicken feed. In the Mediterranean, it is also used as food (cooked) on Korčula island in Croatia [37] and even more commonly on Sicily [38].

*Portulaca oleracea* is a very popular wild vegetable and frequently used cooked or as a raw salad on Sicily and in other Mediterranean regions [37,38]. Our study noted uses for traditional soup with potato, egg omelette, pickled salad, and cooked and served with pasta. 

The use of *Capsella bursa-pastoris* in the raw salad is close to using it like children’s snacks in Poland [39] and Spain [40,41], and in the Adriatic area it is also used cooked in “divlje zelje” mixture [37].

In the Komen area, the following plants served as substitutes for coffee: *Ficus carica, Hordeum vulgare*, and *Quercus pubescens*. Famine food during WWI was *Carpinus betulus leaves*, while its wood was used for the vineyard stakes. *Sambucus nigra* berries wine is also known to locals. Such ways of using these plants have been known since periods of famine and poverty during the last centuries.

*Robinia pseudoacacia* is more frequently used and with more use categories in the Komen area. Traditionally, *R. pseudoacacia* fried flowers are used in a pancake mixture, the same way as documented on Ćićarija [30], while its wood is used for making of garden stakes or musical instruments (flageolets or flutes, horns; local names: “pišćavec” or “frula” and “rog”).

Unlike in the Izola area, some species are in the Komen area used both for food and medicine, e.g., *Iris* sp. tuber is used as potatoes boiled in the peel and as feed to boost the immunity of postpartum cows. Those ways of use are similar to findings on Ćićarija [30]. 

### 4.2. Use of Wild Plants for Food

Today in Europe, we are witnessing the innovation of the traditional use of local wild herbs and finding new ways of their application in modern cuisine [26]. For example, the use of *Aloysia citriodora* in the Adriatic islands is little known, except that it is widely used in Korčula [42]. However, we recorded the use of this plant as food in six cases, suggesting that this is a new trend spreading in the region.

Species like *Borago officinalis*, *Dioscorea communis*, *Iris* sp., *Clematis vitalba*, *Urtica* spp. are usually cooked before eaten due to the presence of thermolabile toxic substances [43], bristly or stinging hairs, or thorns. The use of the five mentioned species is recorded on Sicily, too [38].

In the hinterland of big and rich cities like Trst (Trieste) or Koper (Capodistria), there was no real famine in the last two centuries. The famines were more frequent on the poorer Dalmatian islands and the coast [37], isolated from the big commercial and industrial centres. The development of nearby cities has enabled farmers from Izola and Komen municipalities to improve their quality of life by selling their products like vegetables, meat, wine, oil and even wood to buyers from the cities. However, it is interesting that young shoots of *Arundo donax* are used in the Izola area as a sweetener (substitute for sugar) even today, probably due to the lack of sugar during WW1 and WW2 or among the poorest people.

In the Komen area, an active group of 13 agro-tourism farms have joined together in the “Društvo Planta” association. This group promotes and preserves traditional local gastronomy in combination with “new knowledge”. Thematic local events in the form of festivals are pretty usual in this area (e.g. “Praznik Drijenka”, “Festival Kraška Gmajna”, “The Month of Karst Cuisine”). Nevertheless, we found that some species have been used only recently (mainly on agro-tourism farms and in inns), without any connection to culinary traditions or ethnobotanical use. *Lamium maculatum*, *Bellis perrenis*, *Primula vulgaris*, *Salvia pratensis*, *Sambucus nigra*, *Trifolium pratense* and the flowers of *Viola* sp. are used fresh in salads. These plants are also mentioned by top chefs in Italy who use them in their kitchens [26]. Adding *Sanguisorba minor* and *Satureja hortensis* leaves or *Asparagus acutifolius* shoots to ice cream are also new practices. We can attribute the recent trends to the activities of the mentioned association and the availability of culinary courses and shows on the Internet.

One of the important forces influencing local pharmacopoeias is the presence of local herbal specialists. Such individuals may help maintain the general knowledge of herbs, but it may also have a homogenising effect if their knowledge is influenced by popular literature [42]. An influential local herbal biologist and plant expert, Stipe Hećimović, arrived in the Komen area from Lika (Croatia) during the 1990s. He holds courses and lectures on edible and medicinal wild plants, and a few interviewees mentioned him. His work is one of the reasons for unusual, new knowledge and new fashions recorded during the research in the area of Komen. For example, only in the Komen area, the recent uses of two plants were recorded, which were known in Belarus from the 19th century. These are the use of aerial parts of *Heracleum spondylium* (sour soups and potherb, often dried for winter use) and *Aegopodium podagraria* (soups, potherb, rarely dried for winter use) [44]. Besides Belarus people who use the leaves in soups, people in Sweden use shoots, buds, and flowers [26]. 

Wild fruits make up a large percentage of the plants used. During the World Wars, people ate fresh fruit instead of jam (because of the lack of sugar), and after that jam became a popular way to preserve fruit. Documented are many taxa from which jam was and is made: *Cornus mas*, *Prunus cerasifera* and *Rosa canina* in both areas, *Prunus mahaleb* and *Rubus caesius* only in the Komen region, *Rubus idaeus*, *Sambucus nigra* and *Sorbus domestica* only in the Izola region. Among naturalised and cultivated taxa, *Morus alba*, *M. nigra*, *Ficus carica*, *Diospyrus kaki*, *Prunus persica*, and *Prunus domestica* jams are popular in both regions. *Cydonia oblonga* jam was recorded only in the Izola region. However, other wild fruits are used for other purposes, e.g., distillation of brandy (schnapps).

To date, there have been no local or even regional cookbooks published that would highlight local recipes. The cookbooks include several Mediterranean recipes, but those are taken more broadly, i.e., from Venetian, Istrian or Dalmatian cuisine. 

### 4.3. The Use of Herbs for Medicinal Purposes

Altogether, 72 taxa were reported as used for medicinal uses. For comparison, in a recent study in nearby Ćićarija (Croatia), Vitasović-Kosić et al. [30] reported 90 species being used for medicinal purposes. In comparison, Varga et al. reported 83 species from a study carried out in inland Dalmatia [45]. 

People in the area of Komen use *Gentiana lutea* subsp. *symphyandra* for medical purposes in the form of schnapps infusion, and they consider it good for the liver. In the area of Ćićarija the same preparation is used as anti-gout, anti-rheumatic, and anti-arthritis medicine [30]. *Gentiana* does not grow in Karst Plateou (Kras), but on the nearby mountain of Nanos and Trnovski gozd. Three respondents in the Komen area stated that *Nerium oleander* and *Ruta graveolens* were used to induce abortion. 

In Komen and Izola areas, we recorded the traditional use of *Laurus nobilis* to make an expectorant syrup from its fruits and used to fight coughs and colds. In the same way, syrups (sometimes called pine needles honey) are made from *Picea abies* (anti coughs) and *Pinus* spp. (expectorant). 

*Hedera helix*, an otherwise poisonous plant, is used to make cold syrups. *Plantago lanceolata* is used to treat open wounds, in anti-cough syrup and so-called “trpočo” syrup. For making the “trpočo” syrup, the preparation must be in the ground (“sotto terra”) for three months. Moreover, *Plantago lanceolata* is known as edible raw on a salad.

Some medicinal plants recorded in our research, such as *Parietaria judaica*, *Elymus repens*, *Plantago* spp., and *Ruta graveolens*, are already known to be used for medicinal purposes in the northern Adriatic region [5].

Other examples are rare medicinal uses of *P. judaica* for a urinary tract and *Cynodon dactylon* (L.) Pers. as a purgative, which is still very common in the Adriatic region [34,42].

### 4.4. Practical Use of Plants on Farms and Households

The use of plants for economic purposes in the household or on the farm is not frequent. People used to make stakes and poles from *Robinia pseudoacacia*, *Fraxinus* sp., and *Carpinus betulus*. They tied plants or vines to support using *Clematis vitalba*, *Spartium junceum* (Izola area), and *Salix purpurea* (Komen). 

*Arundo donax* wood is suitable for making flutes, while *Diospyros kaki* wood is good for making tool handles (hoes, pitchforks). 

A recently appeared custom in the area of Komen is making wreaths from various flowers and plants (wild also) for the front doors.

The uses of some plants in the researched area seem entirely forgotten. For example, the use of rough leaf of *Broussonetia papyrifera* (L.) Vent., and *Parietaria judaica* L. for washing bottles and barrels is still very common in the Adriatic area [42], while it was mentioned only one time in the Izola area.

### 4.5. Comparison with Other Research

Since 2010, several ethnobotanical surveys have been conducted in Slovenia and surrounding countries. We compared the results regarding the number of respondents and the number of plant species recorded (Table 4). In addition, based on the available plant lists, JI was calculated to assess the similarity of our results with the results of other studies.

We have to mention that some of the studies we used for comparison were focused on specific categories of use (e.g., medicine or food). Our research covered all plants regardless of the use category. Due to that, the total number of species is smaller in some of the studies compared.

According to the obtained JI, our results have the highest similarity with the results of Lumpert and Kreft (JI = 45.93) for the Karst and Gorjanci area [5]. The Karst area also includes the Komen area, and a comparison of our results for Komen and Lumpert and Kreft’s [5] results for Karst shows a slightly lower similarity (JI = 42.68 %). JI is also higher than 40% for the research of Vitasović-Kosić et al. [30], while the index values for all other studies are lower than 30%. The least agreement is with the survey conducted in Montenegro [46], probably due to major differences in habitats and methodology used. Only Coassini Lokar and Poldini [6] recorded more taxa than in our study among the reviewed studies. However, they covered a larger area with more respondents in their research than in our study.

## 5. Conclusions

Indeed, ethnomedicinal knowledge is not static but evolves according to several elements, such as changes in ecological availability, socioeconomic conditions, and political context on ecological knowledge (borders and different political economies).

However, it should be highlighted—and it is not apparent at first sight—that ecological knowledge, especially on the use of wild plants, appeared in temporal layers (across the time scale). Old, traditional knowledge, based on centuries-old traditions, mixed the school knowledge from 19th and 20th century books and “new knowledge”, derived from mass media as a popular trend of “back to nature” in recent decades. Furthermore, the “layers of knowledge” are additionally mixed by the immigration of people from other parts of Slovenia or internationally. In this case, particularly from the inner parts of Slovenia to the edge of the Mediterranean (both Karst and Istria) due to milder climate and higher living standards. In that light, it is not easy to reveal the source of knowledge, which resembles precise archaeological excavations. 

The divergence in ethnobotanical and ecological knowledge between the two areas studied is considerable. In the Komen area, many people still use wild plants daily for various purposes. In the Izola area, the use of wild plants is limited to a smaller number of people and mainly to the seasonal use of certain plants (for egg omelettes).

We may conclude that the biocultural heritage of Komen municipality is more vital, more homogenous, and connected to their local identity. It reflects in local festivities, associations, and events that maintain the economic importance of wild plants, as well as knowledge and traditions associated with them.

## Figures and Tables

**Table 1:** Ten taxa with the highest relative frequency (RFC) by study areas.

All Respondents		Izola Hinterland		Komen Area	
Taxon	RFC	Taxon	RFC	Taxon	RFC
*Taraxacum officinale ^+^*	0.88	*Asparagus acutifolius*	0.93	*Taraxacum officinale*	0.90
*Asparagus acutifolius ^+^*	0.85	*Taraxacum officinale*	0.87	*Asparagus acutifolius*	0.77
*Foeniculum vulgare ^+^*	0.63	*Rosa canina*	0.77	*Melissa officinalis*	0.77
*Rosa canina*	0.63	*Salvia officinalis*	0.67	*Urtica dioica*	0.73
*Urtica dioica ^+^*	0.62	*Dioscorea communis*	0.63	*Cornus mas*	0.70
*Salvia officinalis*	0.58	*Foeniculum vulgare*	0.60	*Foeniculum vulgare*	0.67
*Laurus nobilis ^+^*	0.55	*Rubus caesius*	0.60	*Sambucus nigra*	0.67
*Sambucus nigra*	0.55	*Laurus nobilis*	0.53	*Laurus nobilis*	0.57
*Cornus mas*	0.52	*Urtica dioica*	0.50	*Thymus longicaulis*	0.57
*Rubus caesius*	0.52	*Ruscus aculeatus*	0.50	*Robinia pseudoacacia*	0.53

^+^ Taxa among 10 most mentioned in both areas.

**Table 2:** Comparative overview of tax by study areas: top five taxa according to frequencies by five use categories (E, M, A, and T).

Izola		Komen	
Taxa	Freq.	Taxa	Freq.
Plants used for food (E)			
*Asparagus acutifolius*	28	*Taraxacum officinale*	27
*Taraxacum officinale*	26	*Melissa officinalis*	23
*Rosa canina*	23	*Asparagus acutifolius*	22
*Dioscorea communis*	19	*Urtica dioica*	21
*Foeniculum vulgare*	18	*Cornus mas*	20
Plants for medicinal use (M)			
*Plantago lanceolata*	11	*Tilia cordata*	13
*Salvia officinalis*	10	*Plantago lanceolata*	10
*Sempervivum tectorum*	8	*Achillea millefolium*	9
*Sambucus nigra*	7	*Sempervivum tectorum*	7
*Urtica dioica*	7	*Salvia officinalis*	7
Plants used in making of alcoholic drinks (A)			
*Ruta graveolens*	10	*Juniperus communis*	9
*Juniperus communis*	5	*Vitis vinifera*	7
*Juglans regia*	5	*Cornus mas*	5
*Vitis vinifera*	4	*Ruta graveolens*	5
*Ziziphus jujuba*	4	*Juglans regia*	5
		*Salvia officinalis*	5
Plants used for making of tools (T)			
*Arundo donax*	6	*Robinia pseudoacacia*	9
*Corylus avellana*	1	*Corylus avellana*	3
*Robinia pseudoacacia*	1	*Quercus pubescens*	2
		*Celtis australis*	2
		*Fraxinus* sp.	2

**Table 3:** Top five species by CV in the sample and subsamples.

	All Respondents		Izola		Komen	
Rank	Taxa	CV		CV	Taxa	CV
1	*Taraxacum officinale*	0.379	*Asparagus acutifolius*	0.427	*Taraxacum officinale*	0.453
2	*Asparagus acutifolius*	0.334	*Salvia officinalis*	0.257	*Urtica dioica*	0.394
3	*Urtica dioica*	0.282	*Taraxacum officinale*	0.239	*Foeniculum vulgare*	0.305
4	*Foeniculum vulgare*	0.277	*Rosa canina*	0.197	*Sambucus nigra*	0.292
5	*Laurus nobilis*	0.225	*Foeniculum vulgare*	0.189	*Robinia pseudoacacia*	0.292

**Table 4:** Comparison with other surveys: total number of plants and plant taxa recorded.

Source	Country	№ of Informants	№ of _Plants	Jaccard Similarity Index
This survey	Slovenia	60	157	
Coassini Lokar and Poldini, 1988 [6]	Italia, Slovenia	67	181	25,.65
Dajić Stevanović et al., 2014 ^1^ [46]	Serbia	-	52	15.47
Dolina et al., 2016, Krk [34]	Croatia	55	76	23.94
Dolina et al., 2016, Poljica [34]	Croatia	67	80	21.54
Ferrier et al., 2015 [22]	Bosnia and Herzegovina	25	58	15.59
Łuczaj and Dolina, 2015 ^2^ [47]	Croatia	49	66 (82)	22.56
Lumpert and Kreft, 2017, Karst [5]	Slovenia	25	77	42.68
Lumpert and Kreft, 2017, Karst and Gorjanci [5]	Slovenia	50	94	45.93
Mattalia et al., 2020 [1]	Ukraina, Romania	61	18	12.70
Menković et al., 2011 [48]	Montenegro	75	94	17.29
Pieroni, 2017 ^2^ [49]	Albania	32	52	20.81
Vitasović-Kosić et al., 2017 [30]	Croatia	50	121	41.84
Žuna Pfeiffer et al., 2020 [22]	Croatia	54	44	20.36

^1^ The study is based on available publications, not on a survey. ^2^ 82 species altogether, 66 mentioned more than one time.

## Data Availability

The data presented in this study are available on request from the corresponding author. The data are not publicly available due to privacy reasons.

## Appendix A1

**Table A1:** The list of documented plant taxa in the Slovenian regions of Komen (Kras) and Izola hinterland (Istria) *.

Botanical Taxon, Family, and ID ZAGR Herbarium	Frequency			Use Category			Local Name	Status	Used Part	Preparation	
	Iz.	Ko.	All	Iz.	Ko.	All				Izola	Komen
*Achillea millefolium* L. (Asteraceae) ZAGR57526	3	14	17	E, M	E, A, M, O	E, A, M, O	rman	W	fl, lf, ap	medicinal infusion for better digestion, stomach problems, hormonally regulates menopause, raw in salad	medicinal infusion, raw in salad or chewing in mouth, mixed in herbal schnapps, in “frtalja”, for wreath on the door
*Actinidia chinensis* Planch. (Actinidiaceae)	1	1	2	E	E	E	kivi	C	fr	fresh fruit, marmalade	fresh fruit
*Aegopodium podagraria* L. (Apiaceae)		5	5		E	E	regačica	W	lf	-	new fashion: boiled in “minestra”, mixed with Urtica sp., like spinach
*Aesculus hippocastanum* L. (Sapindaceae)	3	2	5	A, M	M	A, M	divlji kostanj	W	sd	Maceration in grappa, topical application, for massaging and varicose veins	maceration in wine, topical application for scar healing
*Allium ampeloprasum* L. (Amaryllidaceae)	11	7	18	E, M	E	E, M	česan, divji česan, divji češen, divlja čebula, divlji česan, divlji češan, divlji luk, vinogradarski luk	W	bl, lf	boiled with eggs, medicinal plant	boiled, with scrambled eggs (“frtalja”), in minestrone, risotto
*Allium cepa* L. (Amaryllidaceae)	1	3	4	E, M	E	E, M	čebula	C	bl, lf	for all kind of minestrone, raw with honey anti cough	for all kind of minestrone
*Allium porrum* L. (Amaryllidaceae)	11	5	16	E	E	E	divji por, divlji por, por	W	bl, lf	mixed with Asparagus in “fritaja”, mixed with “palenta”, in minestrone, raw on salad	with scrambled eggs (“frtalja”)
*Allium sativum* L. (Amaryllidaceae)	1	3	4	M	E	E, M	česan, česnik, češnjak	C	bl, lf	against a sore throat—string on a thread and keep a neckles around the neck	in all kinds of dishes, raw for medicinal uses, good for everything
*Allium schoenoprassum* L. (Amaryllidaceae)	1	5	6	E	E	E	drobnjak, vlasac, stoljetna čebula	W/C	lf	raw on salad	raw on salad, in “frtalja”
*Allium ursinum* L. (Amaryllidaceae)	8	2	10	E	E	E	čemaž, divlji čemaž	W	lf	for soup, pie’s, puff pastry	raw salad, soup, pesto, pastry, cake “potica”
*Aloysia citriodora* Palau (Verbenaceae)	2	4	6	E, A	E, A	E, A	citronka, erba luiga, leviš, luviš, erbauliza, limončelo, luviš	C	lf, ap	infusion with lemon	herbal infusion, for rakija travarica mixture
*Althaea officinalis* L. (Malvaceae)		3	3		E, M	E, M	slez	W	fl, lf	herbal infusion	HI for respiratory organs, raw under the teeth, gargle
*Anthyllis vulneraria* (including *A. v.* ssp. *praepropera*) (Fabaceae)		2	2		M	M	ranjak	W	fl	medicinal infusion	medicinal infusion
*Apium graveolens* L. (Apiacece)	1	4	5	E	E	E	celer, selen, šelen, šelin	C	lf	for soup	minestrone with boiled beans
*Armoratia rusticana* P. Gaertn., B. Mey. & Scherb. (Brassicaceae)	1	6	7	E	E, A	E, A	gren, hren, hrin	W/C	fl, lf, rt	raw	raw leaf and root in salad, freshly grated root with teran wine is used a lot on sweet bread
*Arnica montana* L. (Asteraceae)		3	3		E, A, M	E, A, M	arnika	W	fl, ap	-	herbal infusion, schnapps, ointment for skin and wounds or in olive oil against hemorrhoids
*Artemisia absinthium* L. (Asteraceae) ZAGR57654	6	5	11	E, A, M	A, M	E, A, M	pelen, pelin	W	lf, ap	digestive infusion, schnapps, raw chew	medicine for stomach and digestion, schnapps, liquer "pelinkovac"
*Artemisia dracunculus* L. (Asteraceae) ZAGR57494		8	8		E	E	pehtran	C	lf	-	traditional cake “potica” with cottage cheese and tarragon, strudels with tarragon
*Arundo donax* L. (Poaceae) ZAGR57552	13	1	14	E, T, O	O	E, T, O	kanela, kana, trstika	W/C	st	made instruments trumpets or whistles (“trobentice”, “piščale”), for garden stakes, brooms (tied to a cane and cleaned the bread oven), people ate the young stalks and used them as a sweetener for tea, planted against the wind	for instruments (there is no a lot of plants)
*Asparagus acutifolius* L. (Asparagaceae)	28	23	51	E, A, M	E, A	E, A, M	sparoga, šparga, šparha, šparhlja, šparglja, šparinga, divlja šparglja	W	sh	with scrambled eggs (“fritaja”) and bacon, sausage; risotto; in schnapps (2 pc /1 L), medicinal for urinary track	scrambled eggs (“frtalja”) alone or in mixture (Dioscorea, Ruscus), or boiled asparagus on a salad with a hard-boiled egg, risotto: barley and asparagus, or with pasta, schnapps; new fashion: in ice cream, (in past there were not many asparugus)
*Atriplex hortensis* L. (Amaranthaceae)	1	1	2	E	E	E	loboda	W	lf	boiled	boiled
*Avena sativa* L. (Poaceae)	1	1	2	M	E	E, M	oves	C	fl	herbal infusion mixture: Sambucus, Achillea, Urtica, and Avena sativa	HI
*Bellis perennis* L. (Asteraceae) ZAGR57531	1	8	9	E	E, A, M, O	E, A, M, O	marijetica, marjentice, marjetica, violica, mičkice marjetice	W	lf, fl	raw on salad	raw lf and lf in salad, dried for HI antitusic - (anti cough), schnapps, decoration
*Beta vulgaris* L. (Chenopodiaceae)	6	2	8	E	E	E	bledeš, bledež, bleda, blitva	C	lf	boiled	boiled with *Urtica*
*Borago officinalis* L. (Boraginaceae)		4	4		E, AF	E, AF	bograč, borač, borago	W	lf	-	boiled, for minestrone, feed for bees
*Brassica oleracea* L. var. *acephala* (Brassicacaee)	3	3	6	E	E	E	ohrod, vrzota,	C	lf	boiled	boiled
*Brassica oleracea* L. var. *capitata* (Brassicacaee)	1	3	4	E	E, M	E, M	kupus, zelje	C	lf	raw, boiled	list to draw pus from the body
*Brassica rapa* L. (Brassicacaee)	1	6	7	E	E, AF	E, AF	pesa, rumena repa, repa	C	rt	for pickling	for pickling (eated a lot the whole winter), animal feed
*Calendula officinalis* L. (Asteraceae) ZAGR57528	3	2	5	E, M	E, A	E, A, M	ognjič	W/C	fl	HI, medicinal ointment (pork mixed with wax) for skin diseases, against fungus on the feet	HI mixture, medicinal ointment for skin diseases, schnapps
*Cannabis sativa* L. (Cannabaceae)		2	2		O	O	konoplja	C	ap	-	it was once bred to make cloth
*Capsella bursa-pastoris* (L.) Medik. (Brassicaceae) ZAGR57536		2	2		E	E	plešac, plešec	W	lf, fr		raw salad
*Carpinus betulus* L. (Corylaceae)		3	3		T, O	T, O	gaber, gaber, beli grab	W	lf, ap	-	people ate leaf in the first world war, for stakes in the garden and vineyard
*Carum carvi* L. (Apiaceae)	2	2	4	E, M	E, M	E, M	kimel, kumin	W	sd, lf	infused seeds against flatulence in infants; spice, leaves with scrambled eggs	spice, HI
*Castanea sativa* Mill. (Fagaceae)	3	1	4	E	E	E	kostanj, kostanja	W	fr	boiled or baked	boiled or baked
*Celtis australis* L. (Ulmaceae) ZAGR57655	1	5	6	E	E, T, O	E, T, O	koprivca, koprivovec, ladonja	W	fr, ap	raw fruit	raw fruit, wood for hoe and for making whips
*Centaurium erythraea* Rafn (Gentianaceae)	4	1	5	M	A	A, M	milefiori, tauzentrož, tauzentroža, milefiori, taužentroža	W	lf, fl	bitter medicinal HI—good for the stomach, to clean the blood and against stomach pain	the mixture for herbal grappa
*Chelidonium majus* L. (Papaveraceae)		4	4		M	M	krvavi mlačnik, krvavi mlečnik, krvavi mlećak	W	cs		apply cell juice against skin warts or ointment for 1 month until the warts fall off
*Chenopodium album* L. (Chenopodiaceae) ZAGR57652		3	3		E	E	loboda	W	lf	-	boiled like spanach, for soup, stew
*Cicer arietinum* L. (Fabaceae)	1	1	2	E	E	E	čičerika	C	fr	boiled	boiled
*Cichorium intybus* L. (Asteraceae)	11	10	21	E, M	E, M	E, M	cikorija, divji radič, potrošnik, divlji radič, konjski radić	W	rt, lf	dried roasted root coffee substitute—opens digestion, can also be used as HI; root medicinal against diabetes; stew is cooked, raw salad	with scrambled eggs on bacon (was no oil), as a salad, but also cooked with potatoes, anti-diabetic root, root coffee substitute, all roots are eaten in winter sliced and pickled
*Citrus limon* (L.) Burm. f. (Rutaceae)	1	1	2	E, A	E	E, A	limon	C	cs, bk	raw fruit, liqueur limoncello	liqueur limoncello
*Clematis vitalba* L. (Ranunculaceae) ZAGR57488	3	5	8	E, AF	E, AF, O	E, AF, O	sarabot, serabot, srbot	W	sh, ap	young shoots with scrambled eggs	eaten in the past; baskets for mushrooms or leaves (žbrinca) were woven and the vineyard was tied; the dried stem was smoked; as litter—under the animals to be dry and clean
*Clinopodium nepeta* (L.) Kuntze (Lamiaceae)	1	5	6	E	E, M	E, M	divlja meta, divlje origano, divlji oregano, gozdna meta, mačja meta, majoran	W	ap	tea, spice	spice, medicinal infusion
*Cornus mas* L. (Cornaceae) ZAGR57499	10	21	31	E, M	E, A	E, A, M	dren, drenjak, drenjula, drnul, drnjola, drnjul, drnjula	W	fr	marmalade, syrup, raw fruit, medicine against diarrhea	herbal infusion, raw fruit, marmalade, schnapps, liquer, syrup
*Corylus avellana* L. (Betulaceae)	1	5	6	E, T	E, T, O	E, T, O	leska, lešnik, ljeska, rogova, divlji lješnjak	W	sd, ap	nuts raw fruit, for garden stakes	nuts raw fruit, for garden stakes, for children play
*Cotinus coggygria* Scop. (Anacardiaceae)	1	1	2	AF	O	AF, O	ruj, rujevina	W	lf	feed	wreath on the door
*Crataegus monogyna* Jacq (Rosaceae)	7	10	17	E, M, O	E, A, M	E, A, M, O	beli trn, glog, gloh, medvjeđa hruška, medvedova hruška, medvjedja jagoda, medvjeđa hruškica, rdeči gloh, rdeči trn, rjavi trn	W	fr, fl, lf	anti-pressure tea from flowers and leaves, raw fruit, in salad, in the closet against moths	in schnapps and as a liqueur, raw fruit, HI of flower and fruit, good gor heart
*Cydonia oblonga* Mill. (Rosaceae)	3	5	8	E	E, A	E, A	kutina	C	fr	marmalade (mixed with apple)	in schnapps and as a liqueur, marmalade, HI
*Cynara scolymus* L. (Asteraceae)	2	3	5	E	E, M	E, M	artičoka, artičoko	C	fl, lf	boiled with olive oil	boiled with olive oil, leaf HI lowers blood pressure, to cleanse the liver
*Daucus carota* L. (Apiaceae)	1	1	2	E	E	E	korenček, rumen, merlin divlji	W	rt	boiled in minestrone (stew)	boiled in stew (minestrone)
*Dioscorea communis* (L.) Caddick & Wilkin (Dioscoreaceae) ZAGR57540	19	7	26	E	E, A	E, A	blušć, brušlič, blušt, bljušč, bruslič, brusnić, brušlič, blušć, bljušt	W	sh	scrambled or hard boiled eggs, alone or in mixture with Asparagus, on pasta too	scrambled or hard boiled eggs, alone or in mixture with *Asparagus* and *Ruscus*, on pasta too, schnapps
*Diospyros kaki* L.f. (Ebenaceae)	3	3	6	E	E, T	E, T	kaki, zlatna jabuka, pomikaki, kaki	C	fr	raw and dried fruit, marmalade	hoe handle wood; marmalade
*Diplotaxis tenuifolia* (L.) DC. (Brassicaceae) ZAGR57515	14	5	19	E, O	E	E, O	divja rukola, divlja rokula, divlja rukola, divljo rukolo, ruklja, ruklja divlja, rukola	W	lf, fl	on salad alone or with Taraxacum, flower for decoration	raw salad
*Equisetum arvense* L. (Equisetaceae)	1	1	2	M	O	M, O	preslica, preslico	W	ap	prostate HI	against downy mildew, HI keeps fungi at the soil
*Eriobotrya japonica* (Thunb.) Lindl. (Rosaceae)	4	1	5	E	E	E	nešpula, nešpulja	C	fr	raw	raw
*Eruca vesicaria* (L.) Cav. (Brassicaceae) ZAGR57950	3		3	E		E	motovilec, rečica, rukola užitna	C	lf	raw salad	raw salad
*Fagopyrum esculentum* Moench (Polygonaceae)		5	5		E	E	ajda, heljda	C	sd		buckwheat polenta porridge and potica with walnuts
*Ficus carica* L. (Moraceae) ZAGR57491	13	9	22	E, A, M	E, A	E, A, M	divlja figa, figa, fiha, repanca (crna smokva), smokva, smokvo	C	fr, cs	fresh fruit, marmalade, schnapps (“figovac”), opens digestion, cell juice smear against skin warts	fresh fruit, marmalade, schnapps, from dried figs (“petrovača”) a coffee substitute was brewed
*Foeniculum vulgare* Mill. (Apiaceae) ZAGR57539	18	21	39	E, A, O	E, A, M, O	E, A, M, O	divlji kormaček, koromač, komarček, komomač, komorač, komaršek kormač, kromač	W	ap, fr, sd	scrambled eggs, in a mixture for sausages, in schnapps to get a nice smell, stew with potatoes, beans and fennel; when they burned witches they put fennel on the bonfire, so that the burnt flesh would not stink	scrambled eggs with fennel and lemon balm (also traditionally for the first of May, so that you are not eaten by mosquitoes); raw salad, soup, with potatoes, fennel seeds cook in beans; HI from seeds against bloating and abdominal cramps especially for nursing mothers and infants; in schnapps alone or in mixture
*Fragaria vesca* L. (Rosaceae) ZAGR57476	5	2	7	E	E	E	divja jagoda, fragola, gozdna jagoda, jagoda	W	fr	raw fruit, for HI	raw fruit, for HI
*Fraxinus* sp. (Oleaceae)		7	7		T, AF, O	T, AF, O	jesen, jasen	W	ap		garden or vineyard stakes; instruments (whistles, horn); leafes animal feed during summer drought
*Gentiana lutea* subsp. *symphyandra* (Murb.) Hayek (Gentianaceae)		2	2		A, M	A, M	košutnik	W	tb		infused in schnapps, medicinal for liver
*Hedera helix* L. (Araliaceae)		2	2		E, M, AF	E, M, AF	bršljan	W	lf		anti-cold syrup, winter food for goats
*Helianthus tuberosus* L. (Asteraceae)	2		2	E, M		E, M	paparanbus, topinambur	W, INV	fl, tb	flower infusion for energy and sexual power; inulin for diabetics	
*Heracleum sphondylium* L. (Apiaceae)		5	5		E, AF	E, AF	medvedja taca, dežena, medvjedja šapa, medvjeđa šapa	W	lf		pig feed; new fashion: cooking in stew and baking a leaf in a bread
*Hordeum vulgare* L. (Poaceae)	1	5	6	E	E, O	E, O	jačmen, jašmen, ječmen, ješpren, ječan	C	fr	breast milk substitute (baby food): cook barley in shell, add 1/3 cow’s milk, 2/3 water	stew with Phaseolus and meat; roasted for white coffee; they used 250 years long to make the roof
*Humulus lupulus* L. (Cannabaceae)	6	9	15	E	E, A	E, A	divji hmelj, divlji hmelj, hmelj, hmelj divlji	W/C	sh	scrambled eggs alone or in a mixture	scrambled eggs, with pasta, cooked salad, stew, schnapps
*Hypericum perforatum* L. (Hypericaceae)	6	6	12	M	A, M, O	A, M, O	kantarion, santjenževko, sentjanžeuka, sentjanžeuvka, sentjanževka, krv sv. Janeza, sv. Ivan, šentjanževka, šantjanževka, šentjanžeuka, šenjanževka	W	ap	antidepressant, nerve calming HI, St. John's wort oil for rheumatism, for skin and wound healing	smear the skin with oil against psoriasis, in herbal schnapps (a total of 13 plant), wreaths decoration on the door
*Hyssopus officinalis* L. (Lamiaceae)		3	3		E, A, AF	E, A, AF	ožepk, žepek , ožepek, žpk	W/C	lf, fl		HI, schnapps, spice for meat, feed for bees
*Iris* sp. (Iridaceae) ZAGR57661	1	3	4	AF, M	E, M, AF	E, M, AF, O	iris, perunika	W	tb	immunity postpartum cows	young tuber used as (boiled) potatoes in peel, animal feed
*Juglans regia* L. (Juglandaceae) ZAGR57651	5	8	13	E, A, O	E, A, M, O	E, A, M, O	orah, oreh	C	fr	schnapps, liqueur, fresh fruit, for cakes; leaves in the closet to keep moths out	wood for the butt; traditional cake (orehnjača, orehova potica i štruklji), schnapps, ligueur, good for stomack, and against dementia
*Juniperus communis* L. (Cupressaceae)	8	15	23	E, A, M	E, A, M	E, A, M	brin, brinj, brinove jagode, brinj, čepin, čepinj, čopin, brinje, čopinj	W	fr	syrup antibiotic action, schnapps	added to schnapps against diarrhea, juniper oil against parasites in the body, rubbed under the baby’s nose and abdomen for better digestion, anti-herpes oil, urinary problems, antiviral, HI, fruits in venison stew
*Juniperus oxycedrus* L. (Cupressaceae)	2	1	3	E, A	E, A, M	E, A, M	brinkola rjava, brinj, brinj črni	W	fr	to meke Juniper wine	schnapps, raw fruit for pressure regulation
*Lamium maculatum* L. and L. *orvala* L. (Lamiaceae) ZAGR57466		2	2		E	E	mrtva kopriva	W	fl		new fashion: raw flower in salad
*Laurus nobilis* L. (Lauraceae) ZAGR57542	16	17	33	E, M, O	E, M, C, O	E, M, C, O	labrka, lavrika, lomber, lovor, župon, lumbar	C	lf	expectorant syrup, spice	expectorant syrup, HI anti cough and cold, anti asthma, spice for goulash, stew, cabbage, with a figs (against insects); for decoration; consecrated in church when there were no olives
*Lavandula* spp. (Lamiaceae) ZAGR57518	6	7	13	E, A, M, O	E, A, M, O	E, A, M, O	lavanda, sivka	C	fl, lf, sh	calming HI and bath, essential oil (since the Romans), against moths in closets; in golash and on salad	syrup, Teran liqueur with lavender, calming HI, cosmetic: essential oil and hydrolat, edible: Lavandula with *Salvia off., Rosmarinus off., Mentha* sp. fry in butter, whipped cream with lavender and rosemary flowers, wreaths on the door
*Levisticum officinale* W.D.J.Koch (Apiaceae)	2	5	7	E	E, A	E, A	luštrek, ljublja	C	lf, rt	root and leaves for soup	HI, leaf in scrambled eggs, in mixture for schnapps
*Linum usitatissimum* L. (Linaceae)		3	3		AF, M, O	AF, M, O	lan	C	ap		grown for cloth (there was not much of it due to drought); boiled animal feed, after birth (pigs, cows) for digestion, strength and immunity
*Lunaria annua* L. (Brassicaceae)		2	2		O	O	mamine suze	C	ap		for decoration
*Malus sylvestris* (L.) Mill. (Rosaceae)	1	5	6	E	E	E	divja jabolka, divlja jabolka, divlja jabuka,	W	fr	raw fruit	raw fruit, vinegar
*Malva sylvestris* L. (Malvaceae) ZAGR57529	2	4	6	E, M	E, M	E, M	korenika, slezenovec, nalva, slezanj	W	lf, fl	fresh leaf on tooth against toothache, HI	HI, anti cough syrup
*Matricaria chamomilla* L. (Asteraceae)	4	12	16	E, M	E, M	E, M	kamamila, kamilca , kamilica, kamomila, kamomile	W	fl	calming HI	calming HI, ointment, a compress to reduce swelling (together with dung)
*Melissa officinalis* L. (Lamiaceae) ZAGR57533	4	23	27	E	E	E	melisa, srčno zelje, meliso, srčano zelje, srčno zeljce	W	lf	with scrambled eggs, in stew, HI	HI, with scrambled eggs mixed with *Foeniculum* (also traditionally for the first of May, so that you are not eaten by mosquitoes), syrup, raw salad
*Mentha* x *piperita* L. (Lamiaceae) ZAGR57947	5	8	13	E	E, A, M	E, A, M	menta, meta, meta poprena, metvica, paprova meta, poprova meta	W	lf	tea, schnapps	tea, fresh chopped leaves with strawberries
*Mentha* sp. (Lamiaceae) ZAGR5795, ZAGR57953, ZAGR57956	2	3	5	E, A	E, A	E, A	divlja menta, divlja meta, menta	W	lf	tea from 2 Mentha species	tea, liqueur, syrup, redeemed (economic plant)
*Morus alba* L. (Moraceae)	2	5	7	E	E, A	E, A	murva bela, murvica črna, bela, murva črna, bela	W	fr	raw fruit, marmalade, in schnapps	raw fruit, marmalade, schnapps
*Morus nigra* L. (Moraceae)	9	3	12	E, A, AF	E	E, A, AF	murva, murva črna, murvica črna, murva rjava	W	fr	fruit for people and pigs, marmalade	raw fruit, HI, planted by order of the Austro-Hungarian monarchy
*Nerium oleander* L. (Apocynaceae)		2	2		M	M	oleandar, oleander	C	lf		herbal infusion leaf as an abortive agent
*Ocimum basilicum* L. (Lamiaceae)	2	1	3	E	E	E	basilico, bazilika	C	lf	for making pesto	for making pesto and tomato salsa
*Olea europaea* L. (Oleaceae)	9	4	13	E, M, C, O	E, M	E, M, C, O	oljka, maslina, oliva	C	fr, lf, sd	fruit, olive oil edible and against earache, HI—against cholesterol, high blood pressure and arrhythmia, blood cleansing HI (traditionally for 50 years), biofertilizer for the garden (seeds are grounded for b-vitamin), ceremonial-olive branch in the church	HI against high blood pressure, grounded fruit used as spice, medicinal oil
*Origanum majorana* L. (Lamiaceae)	3	4	7	E	E, A	E, A	majaron, majeron, majerun, majoron	C	ap, lf, fl	spice	spice, in goulash, soup, for schnapps, like a fragrance
*Origanum vulgare* L. (Lamiaceae)	2		2	E		E	origano	W	lf	spice	
*Panicum miliaceum* L. (Poaceae)		2	2		E	E	proso	C	sd		in stew
*Phaseolus vulgaris* L. (Fabaceae)	1	2	3	M	E	E, M	fažo, fižo, mahuna	C	fr, sd	the pods are boiled as a healing tea against diabetes	in stew
*Picea abies* (L.) H.Karst. (Pinaceae)	1	2	3	E, M	M	E, M	smreka, smrekovi vršički	W	lf	anti cough syrup (in a jar put a row of young tops, a row of sugar, repeat until the end)	anti cough syrup
*Pinus* spp. (*P. nigra* J.F.Arnold and *P. sylvestris* L.) (Pinaceae)		3	3		M	M	bor	W	lf		expectorant syrup (“honey from pine needles” add lemon)
*Plantago lanceolata* L. (Plantaginaceae) ZAGR57517	11	14	25	E, M	E, M	E, M	oskolisni tropotec, tropotec, trpočo, trpotec, trpotec oskolisni, trputec uzak, uskolisni trpotec, žilica	W	lf	HI, on open and purulent wounds the crushed leaf draws out pus; anti cough syrup ( "trpocchio" syrup—stands for 3 months “soto terra” (underground))	HI, against herpes, with scrambled eggs, raw on salad, raw on open wounds to heal,
*Plantago major* L. (Plantaginaceae) ZAGR57514	5	2	7	E, M	M	E, M	tropotec širokolisni, trpotec, trpotec okruglolisni, trputac	W	lf	raw on salad, raw on open wounds to heal	raw on open wounds to heal
*Portulaca oleracea* L. (Portulacaceae)	2	10	12	E	E	E	pleviu, tuščak , potolak, plezavec, slezen, portulaca, portulak, portulaka, pleveo, potolak, tolščak, ščir, portulec, tuškak, vodeničnik	W	ap, lf, st	boiled	with scrambled eggs, raw salad, pickled salad, boiled salad, with pasta, traditional soup with potato and Portulaca
*Primula vulgaris* Huds. (Primulaceae)	2	10	12	E	E, A	E, A	trobentica, trubentica, arbentica, žuto, jeglič, jaglac	W	fl, lf	raw on salad, HI	raw on salad, HI, in schnapps
*Prunus avium* L. (Rosaceae)	2		2	E, A		E, A	češnja	C	fr	raw fruit, liquer “češnjevec”	
*Prunus cerasifera* L. (Rosaceae) ZAGR57954	5	7	12	E	E	E	amula, cibora, cimbla, cimbora, cimbura, ringlo, divja sliva, divlja šljiva	W	fr	marmalade	marmalade, raw fruit
*Prunus cerasus* var. *marasca* (Rosaceae)	3	5	8	E	E	E	češnja, maraska, višnja, višula	C	fr	raw fruit, for cakes	marmalada, strudel, dried on tea nets
*Prunus domestica* L. (Rosaceae)	9	5	14	E, A	E, A	E, A	čespa, češpa, češplja, sadjevica, šlivovica, sliva, šljiva, tamna šljiva	C	fr	marmalade, schnapps	marmalade, schnapps
*Prunus mahaleb* L. (Rosaceae)		2	2		E, A	E, A	rašelika	W	fl, fr		flower and fruit for liquer Jagermaister, fruit for marmalade
*Prunus persica* (L.) Batsch. (Rosaceae)	3	1	4	E		E	breskva vinogradarska, divjo praska	W	fr	marmalade	marmalade (mixed with apple ar pear instead of pectin)
*Prunus spinosa* L. (Rosaceae)	8	9	17	E, AF	E, A, AF	E, A, AF	brambore, brnauče , brnjovka, brnavča, črni trn, crni trn, trnjula, črn trn, brnjouka, črni gloh, glog na trnu, trnina, trnulj a	W	fr	raw	liqueur, schnapps (10–20 fruit/1 L), cows like to eat it (graze)
*Pyrus communis* L. (Rosaceae)	2	1	3	E, A	E	E, A	hruška, petrovka	C	fr	schnapps	as a substitute for "kom" when roasting schnapps
*Pyrus amygdaliformis* Vill. (Rosaceae)	2	5	7	E	E	E	divije hruškice, divja fruška, divlja hruška, divlja hruškica, divlja kruška, divlja kruškica	W	fr	raw fruit	raw fruit
*Quercus pubescens* Willd. (Fagaceae) ZAGR57479	1	6	7	E	E, T, AF, O	E, T, AF, O	hrast, kraški hrast, želud, žir	W	fr	it was ground to make coffee	to make toys, as coffee substitute, making flour, leaves as litter under domestic animals, feed, for making furniture
*Robinia pseudoacacia* L. (Fabaceae)	4	16	20	E, T	E, M, T, AF, O	E, M, T, AF, O	agacija, ahacija, akacia, gac, akacija, kacia, akacio, hac	W, INV	fl, lf, fr, st	fryng flowers in a pancake mixture traditionally, syrup, for vineyard stakes ("štant")	fryng flowers in a pancake mixture traditionally, for vineyard and garden stakes and for making instruments (“pišćavec”, frula, rog), syrup, leaf feed for piglets when there is a drought, HI flower against rheuma, tree bark as a repellent
*Rosa canina* L. (Rosaceae) ZAGR57523	23	15	38	E, M	E	E, M	pičikul, šipak, pičkul, šipek, srbeuka, srbevka, mimje-mamje, svrbi guzica	W	fl, fr	marmalade, HI against diarrhea, full of c-vitamin, raw fruit	flower on salad, marmalade, HI against diarrhea
*Rosa* cv. (Rosaceae)	2	2	4	E, M, C	E, A	E, A, M, C	kartrose, vrtnica, kartroze	C	fl	medicinal tea, carried to the Church on Corpus Christi	dried petals liqueur, medicinal tea and in “maneštra”
*Rosmarinus officinalis* L. (Rosaceae) ZAGR57519	12	9	21	E, A, M, AF	E, A	E, A, M, AF	rožmarin, ružmarin, roža od morja, rožmarin	C	ap, lf, fl	medicinal tea - to regulate low blood pressure; for circulation, it is added to the bath, to schnapps, spice; for bees very well	into ligueur (for JEGER), spice, schnapps,
*Rubus caesius* L. (Rosaceae) ZAGR57653	18	13	31	E, A, M	E, O	E, A, M, O	kupina, rebida, ribida, robida, robidnica, rubida, rubidnica, rubidenca, rubido, rubidovo perje, rudbenica	W	fr, lf	raw fruit, leaf infusion against colds, liqueur	eating raw fruit, leaf (young leafs) and fruit for herbal infusion, marmalade, in minestrone, young leafs on “frtalja” (scrambled eggs), for colouring the military suit (with dry leafs), the cooperative redeemed
*Rubus idaeus* L. (Rosaceae)	4		4	E		E	malina	C	fr	raw fruit, marmalade	raw fruit
*Rumex acetosa* (Polygonaceae) ZAGR57535	1	5	6	E	E	E	kiselica, kislica	W	lf	raw	raw salad, or cooked
*Ruscus aculeatus* L. (Asparagaceae) ZAGR57492	15	13	28	E, O	E, O	E, O	lobodika, roskula, roškola, roškolo, ruška, ruškola, vaprinac	W	sh, fr, ap	with scrambled eggs or cooked, pickled in jars, graveyard wreaths, like a Christmas tree, a cover for buckets carrying water on donkeys (not to spray water outside)	with scrambled eggs, mixed with *Asparagus* or *Dioscorea* on pasta or rissoto, cream soup, the fruits are ground as a substitute for coffee, or in brine as capers, prosciutto is wrapped in these branches so that insects do not come, for wreaths for the cemetery, young shoots are pickled
*Ruta graveolens* L. (Rutaceae) ZAGR57527	12	6	18	A, M	A, M	A, M	ruda, rudo, vinska rutica	W	ap	good for stomach, in schnapps	cooked with schnapps, leaf tea—abortifacient
*Salicornia* sp. (Chenopodiaceae)	2		2	E		E	salikornia	W	ap	blanched on salad (oil, vinegar)	
*Salix purpurea* L. (Salicaceae)	2	5	7	O	O	O	beka, vrba, venjka	W	st	for knitting "cajne" (round basket), for bottles, “košić”—a small rectangular basket for collecting walnuts	for making"žbrinca" leaf basket; to tie a vineyard
*Salvia officinalis* L. (Lamiaceae) ZAGR57537	20	16	36	E, A, M	E, A, M	E, A, M	salvia, žajbelj, savia, savie, savje, zajbelj, žajbej, žajbel, žajber, žaljbelj	W/C	lf, fl	against sore throat and cough (infusion to which milk can be added), or leaves are added to caramelized milk, to clear catarrh from the lungs 8 days 2x a day chew sage leaves, herbal mixture for schnapps, pasta with sage and garlic	medicinal infusion against cold, syrup against cough, for disinfection, in schnapps, liqueur, spice for venison steak, pesto for pasta, cooked with caramelized milk, syrup from flowers; cooked 15 min, leave 1 day, add lemon, *Mentha* or *Melissa.*
*Salvia pratensis* L. (Lamiaceae) ZAGR57495		2	2		E	E	travniška kadulja	W	fl		new fashoin: raw flower on salad
*Sambucus nigra* L. (Caprifoliaceae) ZAGR57502	13	20	33	E, M	E, A, M, O	E, A, M, O	bazak, bezek, bzg, bezg, bezga, bzek, bzgovin a	W	fl, fr, st	fruit marmalade, fruit or flower syrup, fruit or dried flower herbal tea - against colds; the inflorescence is breaded in a pancake mixture	"wine" of elderberry, jam, syrup and herbal infusion of fruit or flower, liqueur, syrup can be mixed with *Robinia*, the inflorescence is traditionally fried in a pancake mixture, a toy for children (“pušla”) is made of wood, an acorn is placed at the end
*Sanguisorba minor* Scop. (Rosaceae) ZAGR57472		3	3		E	E	strašnica, strašnica zdravilna	W	fl, lf		new fashion: raw flower and leaf on salad, leaf in ice cream
*Satureja hortensis* L. (Lamiaceae)		4	4		E	E	čobar, čubar, čober, čuber	C	lf		spice, new fashion: leaf in ice cream
*Satureja montana* L. (Lamiaceae)	1	8	9	E, M	E, M, O	E, M, O	kraški šetraj, šetraj, ožepek, žepek, žepk	W	lf	spice for meat, infusion against cold	herbal infusion (alone or with *Mentha piperita* and *Melissa*, spice, add for schnapps, soup, stew, ice cream from young leaves, dry leaves grounded into bread mixture, hydrolate as cosmetic, door wreaths they planted and harvested *Satureja*
*Sedum acre* L. (Crassulaceae) ZAGR57662		7	7		C, O	C, O	brdovica, brdovičnik, homulica, rožice sv. Ivana, sv. Ivana rožice	W	ap		tradition on the summer solstice (23/24.6.) woven small crosses placed on the window against spells, to ward off evil spirits (“wreaths of st. John”)
*Sedum telephium* L. ssp. *maximum* (L.) Krock. (Crassulaceae) ZAGR57955	1	4	5	E, M	A, M	E, A, M	hermelika	C	lf, cs	raw for stomach	info schnapps, raw on open wounds
*Sempervivum tectorum* L. (Crassulaceae)	9	8	17	E, M	M	E, M	natresek, natresk, čuvarkuća, ušnik, ušesnik, ušnjak, všesnik, zaušnik	W	lf	raw in salad, against earache - crushed and mixed with olive oil, or the whole piece of the plant in the grill, charred in olive oil and the ear on the pot - to be inhaled.	against earache (cell juice) and for dripping eyes
*Silybum marianum* (L.) Gaertn. (Asteraceae)		4	4		M	M	pegasti badelj	W	sd		herbal infusion restores the liver (seeds are soaked overnight, drunk in the morning) or the seeds are ground into yogurt and eaten
*Sonchus asper* (L.) Hill (Cichoriaceae) ZAGR57532	2		2	E		E	navadna škrbinka, skršeleta	W	lf		boiled with potatos
*Sonchus oleraceus* L. (Cichoriaceae) ZAGR57546	14	1	15	E, AF	E	E, AF	grandačo, grendeču, grendaču, grandič, grendačel, grendačo, grendaučeu, grendeč, grentč, mlečak	W	lf	it is cooked with beans and potatoes, and eaten in winter, the old people used to gather, animal feed	boiled with potatos
*Sorbus aria* (L.) Crantz (Rosaceae)		3	3		E	E	mokovac	W	fr		raw fruit
*Sorbus aucuparia* L. (Rosaceae)	3		3	E		E	brekuja, brekula	W	fr	raw fruit	
*Sorbus domestica* L. (Rosaceae)	11	11	22	E	E, A	E, A	oskoruš, oskoruša, koška, koska, oskorušva, oskurš, oškurš, skurš, škorš, škorša, škorž, škruša, škurš, škurša	W	fr	marmalada	raw fruit, soak in schnapps, liqueur, jam, they also ate unripe (green) fruits
*Spartium junceum* L. (Fabaceae)	2		2	O		O	brnist, žineštra, brništra, žaneštra	W	st	ribbons to tie a vineyard	
*Stellaria media* (L.) Vill. (Caryophyllaceae) ZAGR57496		10	10		E, AF	E, AF	kokošja, kurja creva, kurije črevco, kokošja creva, kurja crevac, kurja čreva, kurja črevca, kurje crevce, kurje crevo, zvezdica	W	ap		traditionally raw on salad, scrambled eggs, for minestre, chicken fed
*Symphytum officinale* L. (Boraginaceae)		3	3		E, M	E, M	gabez, gavez	W	rt		young leaves on salad, roots for medicinal ointment (wound healing)
*Tanacetum parthenium* (L.) Sch. Bip. (Asteraceae) ZAGR57509		11	11		E, M, O	E, M, O	badvujalca, baldrijan, madarijolca, maderjana, madrjolca, mandrijanca, mandrjanca, madarijolca, mdrjanca, madrjanca	C	lf		fresh or dried for medical infusion, for prostate and female ailments (against menstrual pain), antidepressant, relaxes, with scrambled eggs (alone or with fennel and lemon balm), flowers to the cemetery
*Taraxacum officinale* F. H. Wigg. (Cichoriaceae) ZAGR57467	26	28	54	E, M	E, A, M	E, A, M	pazdonkolo, jajčar, pizdonkola, pizdunduka, pzdonkola, pzdunkola, radička, smolička, regrat, regrad, pzdunkula, smolčika, pzdonkolo, pizdonkula, jajčer, škržožupca, radičkona, žetenja, radičkovna, rehrat, žetena, žutenica	W	lf, fl, rt	raw salad, blanched with hot beans or/and potatoes, or cooked like spinach, with scrambled eggs, dandelion honey traditionally for coughs and to boost immunity, medicinal infusion of dried flowers, root - against cancer	medicinal infusion for respiratory system, raw on salad or cooked with potatoes, of flower honey and wine (Tolmin recipe), syrup traditional recipe; 20 flowers, 2 L sugar, 2 L water, liqueur from flower
*Thymus longicaulis* C. Presl (Lamiaceae) ZAGR57549, ZAGR57660	5	17	22	E, M	E, A, M	E, A, M	materina dušica, majčina dšica	W	lf, fl, ap	spice, medicinal infusion against tonsillitis	medicinal infusion, goulash, schnapps, spice, scrambled eggs with flowers
*Thymus vulgaris* L. (Lamiaceae)		5	5		E, A, M	E, A, M	timijan, timjan	W	lf, fl, ap		medicinal infusion, liqueur, spice for fresh salad, schnapps mixture
*Tilia platyphyllos* Scop. (Malvaceae)	5	2	7	M	M	M	lipa, navadna lipa	W	lf	good for the heart	good for the heart
*Tilia cordata* Mill. (Malvaceae)		13	13		M	M	lipa, lipo, lipovec	W	lf		medical infusion, large amounts are not good for the heart, anti-fever, syrup
*Trifolium pratense* L. (Fabaceae)		4	4		E, A	E, A	črna detelja, lučerna, detelja, zajča detelja	W	lf		white and red leaves on salad, herbal infusion, liqueur
*Triticum vulgare* L. (Poaceae)	1	2	3	E	E, C, O	E, C, O	šenica	C	ap	tradition at the baptism of a child: godfathers bring bread	tradition at the baptism of a child: godfathers bring big bread, and the first time you see a child-baby, take it by the nose—for happiness and health; once cultivated, today *T. speltum* is cultivated
*Urtica dioica* L. (Urticaceae) ZAGR57543	15	24	39	E, M	E, M, AF, O	E, A, M, AF, O	kopriva, koprva	W	lf	fresh as a salad, cooked with potatoes, in "štruklji", prepared as spinach with cream, medical infusion for blood purification, for prostate, diuretic, anti-rheumatic to strengthen the hair, in the World War I made uniforms of nettle	raw, cooked as spinach, scrambled eggs, for gnocchi, in minestrone, raw as feed for pigs and chickens, medical infusion, anti-rheumatic, against parasites in the garden (macerated solution for 15 days), biofertilizer, for coloring eggs—young tops cooked in wine teran
*Vaccinium myrtillus* L. (Ericaceae)	5		5	E		E	borovnica	W	fr	raw fruit	
*Valeriana officinalis* L. (Caprifoliaceae)	1	3	4	M	M	M	baldrijan	W	lf	medicinal infusion	medical infusion for relaxation, for better sleeping
*Verbascum thapsus* L. (Scrophulariaceae) ZAGR41795		5	5		M	M	lučnik, ločnik, svečnik, škofova kapa	W	lf		medicinal infusion of flowers and leaves for cough, for lungs for catarrh clean
*Verbena officinalis* L. (Verbenaceae)		2	2		M	M	sporiš	W	lf		medical infusion cleanses the liver, spleen
*Viola* sp. (Violaceae) ZAGR57522, ZAGR57538	4	8	12	E, M	E, A, O	E, A, M, O	mačeha, viola, violica, violice	W	fl	medical infusion to regulate blood pressure, to cleanse the blood (often drank when they were children), the flowers are added to the pancake mixture	flower on salad, dried flower for tea, in schnapps, for decoration
*Viscum album* L. (Santalaceae)	1	3	4	A, M	M	A, M	bela imela, bela amela, omela bela	W	lf, fr, ap	medicinal schnapps	the leaf is dried, the green berries are boiled, the decoction maintains the pressure; tincture to regulate blood pressure
*Vitis vinifera* L. incl. *Vitis vinifera* L. ssp. *vinifera* (Vitaceae) ZAGR57489	12	12	24	E, A, M, AF	E, A, M, O	E, A, M, AF, O	loza, trta, drupina, fragola, malvazija, refošk, teran, terano, tropina, trta izabela, trupina, vinova loza	W/C	fr	for making wine, vinegar and schnapps, schnapps wraps to lower body temperature, leaves as food for goats (they like to eat)	for wine, schnapps (several grape varieties: fragola, refošk, malvasia, isabella, teran) and teranino liqueur, as a mixture for schnapps: *Achillea, Calendula* and *Chamomile*, chicken eggs for Easter are colored in Teran wine, for disinfection of open wounds
*Zea mays* L. (Poaceae)	1	3	4	E	E, O	E, O	kuruza, kuruzo, kuruza	W	lf, fr	minestrone	mattresses were stuffed with corn leaves (or sheep wool), minestrone
*Ziziphus jujuba* Mill. (Rhamnaceae)	8	1	9	E, A	A	E, A	žižola, žižula, žižolo	W	fr	raw fruit, schnapps	raw fruit, schnapps

* Legend: Study area: Iz.—Izola, Ko.—Komen. Use categories: E—food or drink, A—alcoholic drinks, M—medicinal use, T—tool, C—ceremonial use, AF—animal feed, and O—other not specified ways of use. Status: W—wilde, C—cultivated, INV). Part used: ap—erial parts, bd—buds, bk—bark, bl—bulb, cs—cell sup, fj—fruit juice, fl—flowers, fr—fruit, im—immature fruits, lf—leaves, lg—legumes, nd—needles, p—peel, po—pollen, pe—petals, pcl—pedicels, pc—pericarp, rs—resin, rt—roots, sd—seed, sh—shoots, st—stalk, sty—stylus, tb—tuber, trt—taproots; u—underground parts, wh—whole plant.

**Table A2:** Cultural Value Coefficient (CV) of the recorded taxa for the whole sample and per study area.

Taxa	All	Izola	Komen
*Achillea millefolium*	0.065	0.004	0.178
*Actinidia chinensis*	0.000	0.000	0.000
*Aegopodium podagraria*	0.001		0.004
*Aesculus hippocastanum*	0.002	0.004	0.001
*Allium ampeloprasum*	0.027	0.042	0.008
*Allium cepa*	0.002	0.001	0.001
*Allium porrum*	0.010	0.019	0.004
*Allium sativum*	0.001	0.000	0.001
*Allium schoenoprassum*	0.001	0.000	0.004
*Allium ursinum*	0.004	0.010	0.001
*Aloysia citriodora*	0.003	0.001	0.005
*Althaea officinalis*	0.001		0.004
*Anthyllis vulneraria* (including *A. v.* ssp. *praepropera*)	0.000		0.001
*Apium graveolens*	0.001	0.000	0.003
*Armoratia rusticana*	0.004	0.000	0.011
*Arnica montana*	0.002		0.009
*Artemisia absinthium*	0.021	0.026	0.011
*Artemisia dracunculus*	0.003		0.010
*Arundo donax*	0.025	0.087	0.000
*Asparagus acutifolius*	0.334	0.427	0.168
*Atriplex hortensis*	0.000	0.000	0.000
*Avena sativa*	0.000	0.000	0.000
*Bellis perennis*	0.014	0.000	0.046
*Beta vulgaris*	0.003	0.006	0.001
*Borago officinalis*	0.002		0.006
*Brassica oleracea*	0.001	0.001	0.001
*Brassica oleracea* var. *capitata*	0.002	0.000	0.004
*Brassica rapa*	0.004	0.000	0.013
*Calendula officinalis*	0.004	0.005	0.001
*Cannabis sativa*	0.000		0.001
*Capsella bursa-pastoris*	0.000		0.001
*Carpinus betulus*	0.000		0.001
*Carum carvi*	0.002	0.002	0.001
*Castanea sativa*	0.001	0.001	0.000
*Celtis australis*	0.007	0.000	0.021
*Centaurium erythraea*	0.002	0.003	0.000
*Chelidonium majus*	0.001		0.003
*Chenopodium album*	0.000		0.001
*Cicer arietinum*	0.000	0.000	0.000
*Cichorium intybus*	0.040	0.045	0.035
*Citrus limon*	0.000	0.001	0.000
*Clematis vitalba*	0.009	0.003	0.014
*Clinopodium nepeta*	0.002	0.000	0.006
*Cornus mas*	0.133	0.035	0.167
*Corylus avellana*	0.006	0.001	0.014
*Cotinus coggygria*	0.000	0.000	0.000
*Crataegus monogyna*	0.059	0.033	0.057
*Cydonia oblonga*	0.005	0.001	0.008
*Cynara scolymus*	0.003	0.001	0.005
*Daucus carota*	0.000	0.000	0.000
*Dioscorea communis*	0.054	0.057	0.016
*Diospyros kaki*	0.003	0.001	0.004
*Diplotaxis tenuifolia*	0.030	0.067	0.004
*Equisetum* sp.	0.000	0.000	0.000
*Eriobotrya japonica*	0.001	0.003	0.000
*Eruca sativa*	0.000	0.001	
*Fagopyrum esculentum*	0.001		0.004
*Ficus carica*	0.073	0.118	0.026
*Foeniculum vulgare*	0.277	0.189	0.305
*Fragaria vesca*	0.002	0.004	0.001
*Fraxinus* sp.	0.006		0.023
*Gentiana lutea* ssp. *symphyandra*	0.000		0.001
*Hedera helix*	0.001		0.003
*Helianthus tuberosus*	0.000	0.002	
*Heracleum spondyllium*	0.002		0.008
*Hordeum vulgare*	0.003	0.000	0.010
*Humulus lupulus*	0.018	0.006	0.026
*Hypericum perforatum*	0.020	0.006	0.023
*Hyssopus officinalis*	0.002		0.007
*Iris* sp.	0.003	0.001	0.006
*Juglans regia*	0.050	0.026	0.066
*Juniperus communis*	0.090	0.042	0.157
*Juniperus oxycedrus*	0.002	0.002	0.001
*Lamium maculatum*	0.000		0.001
*Laurus nobilis*	0.225	0.145	0.259
*Lavandula angustifolia*	0.035	0.034	0.036
*Levisticum officinale*	0.005	0.001	0.011
*Linum usitatissimum*	0.002		0.007
*Lunaria annua*	0.000		0.001
*Malus sylvestris*	0.001	0.000	0.004
*Malva sylvestris*	0.004	0.003	0.006
*Matricaria chamomilla*	0.027	0.008	0.057
*Melissa officinalis*	0.029	0.003	0.084
*Mentha piperita*	0.023	0.004	0.038
*Mentha* sp.	0.003	0.002	0.004
*Morus alba*	0.003	0.001	0.006
*Morus nigra*	0.021	0.051	0.001
*Nerium oleander*	0.000		0.001
*Ocimum basilicum*	0.000	0.001	0.000
*Olea europaea*	0.043	0.091	0.006
*Origanum majorana*	0.004	0.001	0.006
*Origanum vulgare*	0.000	0.001	
*Panicum miliaceum*	0.000		0.001
*Phaseolus vulgaris*	0.001	0.000	0.001
*Picea abies*	0.001	0.001	0.001
*Pinus nigra*	0.000		0.001
*Plantago lanceolata*	0.065	0.056	0.076
*Plantago major*	0.005	0.011	0.001
*Portulaca oleracea*	0.006	0.001	0.016
*Primula veris vulgaris*	0.011	0.001	0.032
*Prunus avium*	0.000	0.002	
*Prunus cerasifera*	0.005	0.004	0.006
*Prunus cerasus* var. *marasca*	0.003	0.001	0.004
*Prunus domestica*	0.013	0.029	0.004
*Prunus mahaleb*	0.000		0.002
*Prunus persica* var. *platycarpa*	0.000	0.001	0.000
*Prunus spinosa*	0.042	0.020	0.056
*Pyrus communis*	0.001	0.002	0.000
*Pyrus amygdaliformis*	0.002	0.001	0.004
*Quercus pubescens*	0.010	0.000	0.030
*Robinia pseudoacacia*	0.107	0.005	0.292
*Rosa canina*	0.127	0.197	0.036
*Rosa* cv.	0.004	0.004	0.002
*Rosmarinus officinalis*	0.087	0.122	0.029
*Rubus caesius*	0.167	0.171	0.058
*Rubus idaeus*	0.001	0.003	
*Rumex acetosa*	0.001	0.000	0.004
*Ruscus aculeatus*	0.073	0.086	0.062
*Ruta graveolens*	0.026	0.046	0.011
*Salicornia* sp.	0.000	0.001	
*Salix purpurea*	0.002	0.001	0.004
*Salvia officinalis*	0.208	0.257	0.164
*Salvia pratensis*	0.000		0.001
*Sambucus nigra*	0.220	0.078	0.292
*Sanguisorba officinalis*	0.000		0.001
*Satureja hortensis*	0.001		0.003
*Satureja montana*	0.013	0.001	0.038
*Sedum acre*	0.003		0.013
*Sedum telephium* ssp. *maximum*	0.004	0.001	0.005
*Sempervivum tectorum*	0.022	0.029	0.008
*Silybum marianum*	0.001		0.004
*Sonchus asper*	0.000	0.001	
*Sonchus oleraceus*	0.019	0.067	0.000
*Sorbus aria*	0.000		0.001
*Sorbus aucuparia*	0.000	0.001	
*Sorbus domestica*	0.040	0.019	0.050
*Spartium junceum*	0.000	0.001	
*Stellaria media*	0.010		0.038
*Symphytum officinale*	0.001		0.004
*Tanacetum parthenium*	0.020		0.091
*Taraxacum officinale*	0.379	0.239	0.453
*Thymus longicaulis*	0.073	0.010	0.178
*Thymus vulgaris*	0.005		0.019
*Tilia platyphyllos*	0.002	0.004	0.001
*Tilia cordata*	0.007		0.027
*Trifolium pratense*	0.002		0.008
*Triticum vulgare*	0.000	0.000	0.001
*Urtica dioica*	0.282	0.105	0.394
*Vaccinium myrthillus*	0.001	0.004	
*Valeriana officinalis*	0.001	0.000	0.001
*Verbascum thapsus*	0.001		0.004
*Verbena officinalis*	0.000		0.001
*Viola arvensis*	0.023	0.005	0.030
*Viscum album*	0.002	0.001	0.003
*Vitis vinifera*	0.111	0.102	0.076
*Zea mays*	0.002	0.000	0.004
*Ziziphus jujuba*	0.009	0.028	0.000
